# Reprogramme the *E. coli* metabolism by engineering a functional carbon-fixation pathway

**DOI:** 10.1186/s13036-025-00612-x

**Published:** 2025-12-29

**Authors:** Yu Chen, Adam Burke, Vincent Chriscoli, Mengru Yang, Ping Chang, Tianpei Li, Buke Zhang, Royston Goodacre, Lu-Ning Liu

**Affiliations:** 1 Department of Biochemistry, Cell and Systems Biology, Institute of Systems, Molecular and Integrative Biology, University of Liverpool, Liverpool, L69 7ZB UK; 2 Centre for Metabolomics Research, Department of Biochemistry, Cell and Systems Biology, Institute of Systems, Molecular and Integrative Biology, University of Liverpool, Liverpool, L69 7ZB UK; 3 MOE Key Laboratory of Evolution and Marine Biodiversity, Frontiers Science Center for Deep Ocean Multispheres and Earth System & College of Marine Life Sciences, Ocean University of China, Qingdao, 266003 China

**Keywords:** Carbon fixation, CO_2_-concentrating mechanisms, *E. coli*, Metabolic profiling, Synthetic biology

## Abstract

**Background:**

Rising atmospheric CO₂ levels and their impact on climate change have intensified the need for innovative carbon capture and fixation strategies. The Calvin-Benson-Bassham (CBB) cycle, a central metabolic pathway in all photoautotrophic organisms and many autotrophic bacteria, plays a pivotal role in global carbon assimilation but is limited by the low catalytic efficiency of Rubisco.

**Results:**

Here, we engineered a complete, functional CBB cycle in *Escherichia coli*, by heterologously expressing up to 13 genes encoding phosphoribulokinase, α-carboxysomes, and inorganic carbon pumps. This bioengineering approach allowed *E. coli* to utilize atmospheric CO_2_ and led to increased levels of sugars such as ribose (4.94-fold) and xylitol (8.94-fold). Detailed metabolomic profiling of central carbon metabolism using gas chromatography-mass spectrometry (GC-MS) demonstrated that installation of the CBB cycle has a notable impact on the metabolic landscape of *E. coli*, resulting in substantial alterations in central carbon and amino acid metabolism. These findings deepen our understanding of the natural biological carbon-fixation pathway and its engineering in heterotrophic hosts. Furthermore, this work provides a versatile platform for evaluating and selecting efficient carbon-fixation modules, as well as assessing metabolic bottlenecks in engineered systems.

**Conclusion:**

These advances offer practical guidance for rational metabolic engineering in diverse organisms for biotechnological applications, including carbon sequestration, sustainable bioproduction, and crop improvement.

**Supplementary Information:**

The online version contains supplementary material available at 10.1186/s13036-025-00612-x.

## Introduction

The greenhouse effect is currently leading to global warming on Earth, which in turn raises worldwide issues like climate change, ecosystem disruptions, and more frequent extreme weather events [1, 2]. As CO₂ emissions continue to rise, achieving carbon reduction and neutrality has become a shared priority for people around the world. In this context, autotrophic carbon fixation pathways are crucial, as they transform CO_2_ into organic carbon and play a significant role in efforts to lower atmospheric carbon levels [3, 4].

The Calvin-Benson-Bassham (CBB) cycle is a natural carbon fixation pathway prevalent across diverse organisms, making a substantial contribution to global carbon assimilation [3, 5]. Ribulose 1,5-bisphosphate carboxylase/oxygenase (Rubisco), the most abundant protein on Earth, is the central enzyme in the CBB cycle, accounting for ~ 95% of inorganic carbon (Ci) fixation [6, 7]. However, Rubisco has evolved as an insufficient enzyme with slow carboxylation rates and low specificity between CO_2_ and O_2_, leading to low CO_2_-fixation efficiency [7–9]. To address these limitations, natural organisms have developed CO_2_-concentrating mechanisms (CCMs) to elevate CO_2_ levels around Rubisco, thereby enhancing carboxylation and suppressing oxygenation [10]. In cyanobacteria and some chemoautotrophs, the CCM employs Ci import systems and specialised proteinaceous microcompartments called carboxysomes [11, 12]. The Ci pumps accumulate bicarbonate within the cytoplasm [13, 14]. The carboxysome encapsulates two key enzymes: Rubisco and carbonic anhydrase (CA) [15, 16]. Accumulated bicarbonate can pass across the semi-permeable carboxysome shell and is then converted to CO_2_ by CA, generating a high concentration of CO_2_ around Rubisco to enhance its carboxylation activity [7, 17, 18].

Apart from natural organisms that are capable of performing carbon fixation, engineering functional carbon-fixing pathways into model heterotrophic hosts provides a powerful approach for unravelling the natural process [19, 20] and developing advanced carbon-fixing systems with potential applications in carbon capture and bioproduction of valuable chemicals [21–24]. The inherent pentose phosphate pathway (PPP) in heterotrophs contains almost all the enzymes and catalytic steps required for a CBB cycle, except for two key enzymes: phosphoribulokinase (Prk), which catalyzes the phosphorylation of ribulose-5-phosphate (Ru5P) to ribulose-1,5-bisphosphate (RuBP), and Rubisco, which fixes CO₂ onto RuBP to form 3-phosphoglycerate (3PG). Incorporation of these two enzymes has enabled the reconstitution of a functional CBB cycle in organisms like *Escherichia coli* and yeast [19, 25–27]. When further supplemented with formate dehydrogenase and optimized via laboratory evolution, engineered *E. coli* have demonstrated the capacity to generate biomass from CO_2_ under high CO_2_ conditions [19]. Moreover, Flamholz, Dugan [28] demonstrated the feasibility of engineering carbon fixation in *E. coli* by integrating the CBB cycle with a carboxysome-based CCM, including a Ci pump (DabA1/B1) and α-carboxysomes. Despite these advances, constructing efficient carbon-fixation pathways requires optimization of the catalytic activities of introduced components and their functional coordination in new hosts [25, 29, 30]. Additionally, comprehensive metabolic profiling, in addition to phenotypic and functional analyses, is essential for examining the metabolic effects of installing the CBB cycle and CCMs and identifying bottlenecks to guide further optimization of metabolomic pathway engineering [31–33].

In this study, we engineered a functional CBB cycle into *E. coli* through incorporation of a bacterial CCM, composed of α-carboxysomes and Ci pumps, along with Prk. This platform allowed us to assess systematically the activities of various Ci pumps in the engineered carbon-fixation system. Using gas chromatography-mass spectrometry (GC-MS), we conducted a comprehensive metabolic profiling analysis of the engineered *E. coli* strains, whereby GC-MS using methoxymation and silylation derivatisation chemistry allows central carbon and nitrogen metabolism to be readily assessed. Our results revealed distinct metabolic shifts triggered by the installation of the carboxysome-based CCM and the CBB cycle. Our findings offer valuable insights into the bioengineering of functional bacterial CCM and the CBB cycle in non-native hosts, and may inform future strategies to optimise biological carbon fixation in engineered heterotrophs to boost carbon assimilation and production of high-value chemicals.

## Results and discussion

### Installation of active Ci pumps

**Fig. 1:**
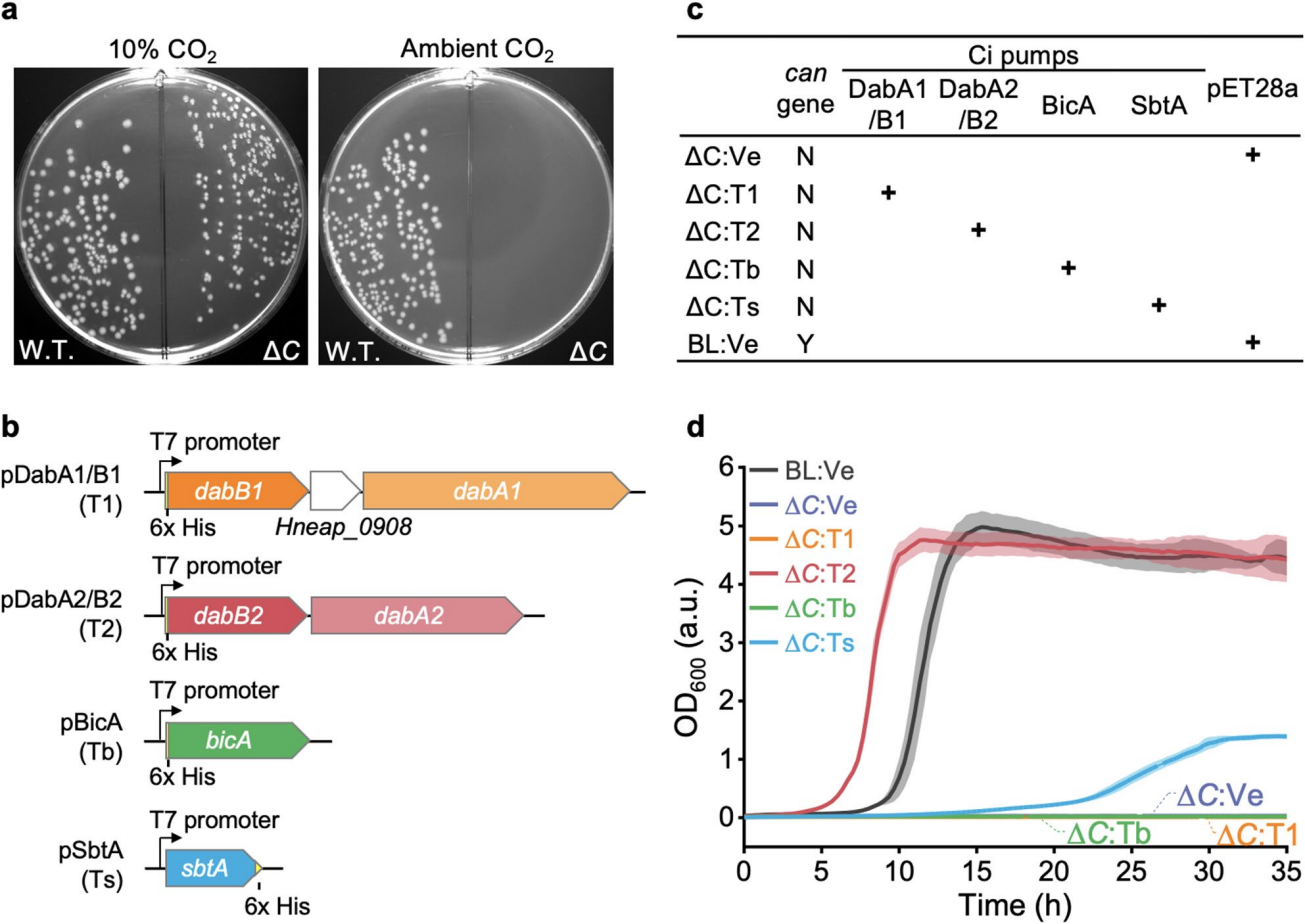
Evaluation of the activity of bicarbonate transporters. (**a**) Demonstrating the high CO_2_ dependency of the Δ*C* mutant. (**b**) shows the plasmids constructed for expressing Ci pumps in *E. coli*. Abbreviations in brackets were used to indicate the expression plasmids in the variants (e.g. Δ*C*:T1 represents the Δ*C* mutant strains harbouring pDabA1/B1). (**c**) shows the strains with different Ci pumps or an empty plasmid incorporated. ‘N’ signifies the absence of the *can* gene, while ‘Y’ indicates its presence. ‘+’ symbols indicate the presence of corresponding Ci pumps or empty plasmid. (**d**) Growth curves of Δ*C* mutant with the presence of different Ci pumps or empty pET28a; LB medium supplemented with 50 µg mL^− 1^ kanamycin, and 0.5 mM isopropyl β-D-1-thiogalactopyranoside (IPTG) was used; a.u.: absorption unit. The solid lines represent the average OD_600_ from 6 replicate inoculations, and the transparent shade along the lines with a similar colour represents the standard deviation

To construct a functional CCM, we first deleted the endogenous CA-encoding gene (*can*) from the chromosome of *E. coli* to generate a high CO_2_-requiring *E. coli* mutant (Δ*C*), in which intracellular bicarbonate must be complemented through a higher environmental CO_2_ level or additional Ci pumps [13, 34] (Fig. 1a). Using this Δ*C* mutant, we assessed the expression and activities of four different Ci-import systems, including DabA1/B1 and DabA2/B2 from *Halothiobacillus neapolitanus* [35], as well as BicA and SbtA from *Synechocystis* sp. PCC 6803 [13, 14]. Functional Ci pumps can supply alternative CO_2_/bicarbonate sources, which are expected to restore the growth of the Δ*C* mutant under ambient air conditions [13, 34], containing ~ 0.04% CO_2_.

Heterologous expression of these individual Ci importers fused with Hexahistidine tags (6xHis-tags) was verified by sodium dodecyl sulfate-polyacrylamide gel electrophoresis (SDS-PAGE) and immunoblot analysis (Fig. 1b, Fig. S1). Growth assays revealed that the Δ*C* mutant expressing DabA2/B2 or SbtA exhibited growth under ambient air conditions (Fig. 1c and d), indicating the robust Ci-importing activities of engineered DabA2/B2 and SbtA. Notably, the DabA2/B2-expressing strain exhibited a higher growth rate than the SbtA-expressing mutant (Fig. 1c and d), suggesting that DabA2/B2 mediates more efficient Ci import than SbtA in the engineered *E. coli* background. In contrast, mutants expressing DabA1/B1 or BicA were unable to grow under ambient air conditions (Fig. 1c and d), although DabA1/B1 and BicA function as Ci pumps in *H. neapolitanus* and *Synechocystis* 6803, respectively [14, 35, 36]. This suggests that their inadequate (or low) Ci-importing activities were unable to meet the bicarbonate requirements of the Δ*C* mutant. These results are consistent with previous studies indicating that DabA2/B2 exhibited the highest Ci-importing activity relative to DabA1/B1 and SbtA [35], whereas engineered BicA was ineffective in restoring the growth of high CO_2_-requiring strains [34].

### Integration of metabolic modules to generate a carboxysome-based CCM and a CBB cycle

**Fig. 2:**
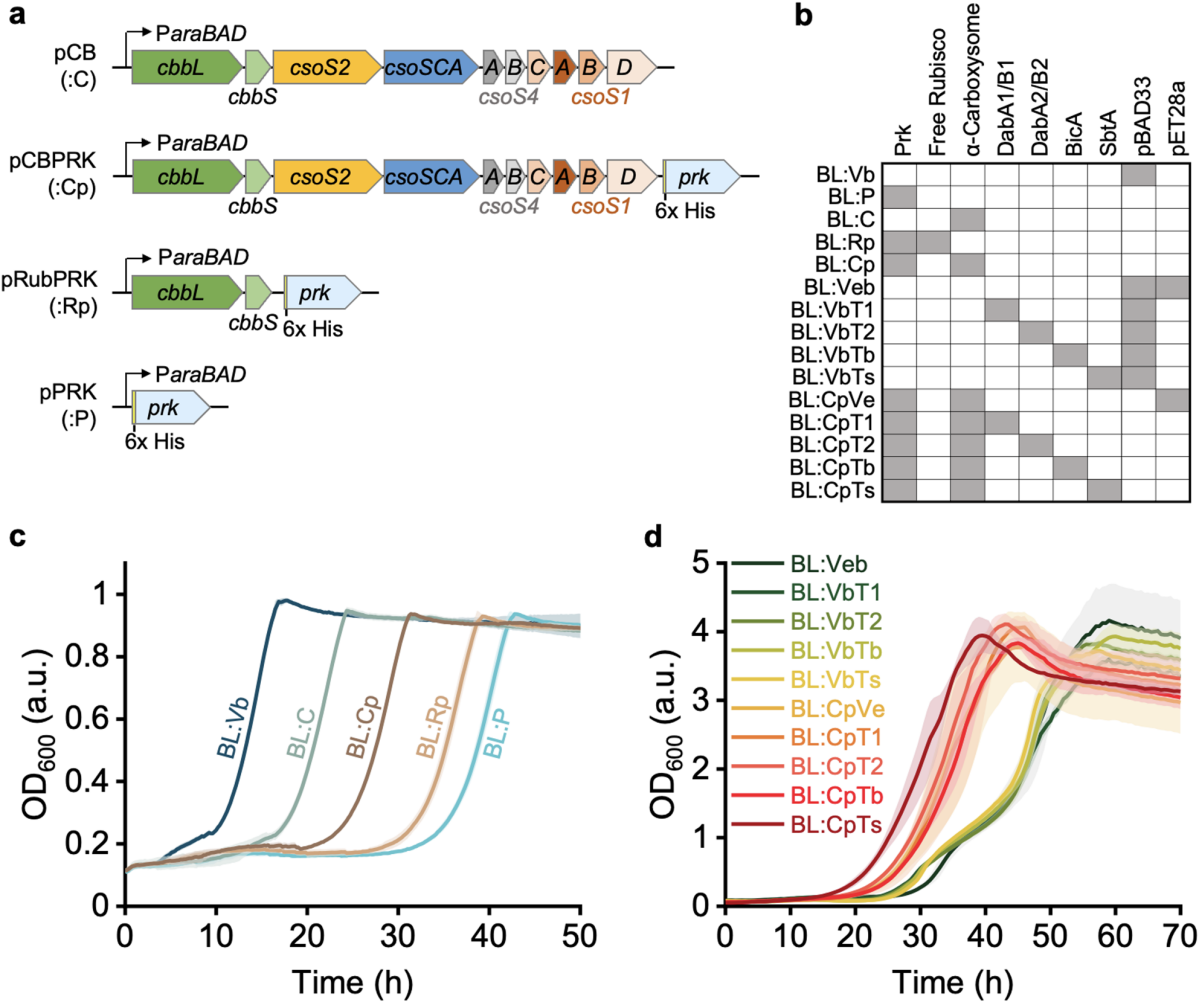
Growth profile of the strains expressing functional recombinant proteins**.** (**a**) Expression plasmids constructed for α-carboxysomes expression (pCB), Prk expression (pPRK), α-carboxysomes and Prk co-expression (pCBPRK), and Rubisco and Prk co-expression (pRubPRK). Abbreviations in brackets are used to indicate the expression plasmids in the variants. (**b**) Strains that possess corresponding components or empty plasmids are labelled using abbreviations given in the plasmid map and indicated by grey boxes in the table. ‘Free Rubisco’ refers to Rubisco expressed alone, without other α-carboxysome components, whereas α-carboxysome represents the carboxysome including the Rubisco and CA encapsulated. (**c**) Growth curves of *E. coli* with the presence of different components of the CBB cycle indicate the functionality of these components. The solid lines represent the average OD_600_ from 6 replicate inoculations, and the transparent shade along the lines with a similar colour represents the standard deviation. M9 medium supplemented with 0.2% glycerol, 50 µg mL^-1^ chloramphenicol, and 1 mM arabinose was used for the growth curves. (**d**) Growth curves of variants harbouring various Ci pumps along with α-carboxysomes and Prk, or the empty plasmid pBAD33 (:Vb). M9 medium supplemented with 0.4% glycerol, 50 µg mL^-1^ chloramphenicol, 50 µg mL^-1^ kanamycin, 1 mM arabinose, and 0.5 mM IPTG was used for the growth curves

To establish a functional CBB cycle, we first generated a plasmid pCBPRK to express the genes for both α-carboxysomes and Prk in *E. coli* (Fig. 2a), which was confirmed by gene sequencing. As a control, we co-expressed Rubisco derived from *H. neapolitanus* and Prk using the plasmid pRubPRK. Expression of these components was validated by SDS-PAGE and immunoblot analysis (Fig. S2).

The growth inhibitory phenotype caused by expression of Prk alone was assessed to validate the functionality of recombinant α-carboxysomes or Rubisco. Specifically, expression of Prk alone directs the carbon metabolism pathway towards RuBP, creating a metabolic dead-end that suppresses host strain growth (Fig. S3). In contrast, a functional CBB cycle (catalysed by Rubisco) could rescue this inhibition through RuBP carboxylation to 3PG (Fig. S3) [21, 37]. When grown in M9 medium supplemented with 0.2% glycerol, the *E. coli* variant expressing Prk only (BL:P) exhibited a substantially prolonged lag phase relative to the BL:Vb control that expresses empty pBAD33 vector (Fig. 2b and c). In contrast, the BL:Rp variant co-expressing Rubisco and Prk displayed enhanced growth with a shorter lag phase; co-expressing α-carboxysomes with Prk (BL:Cp) further improved cell growth (Fig. 2b and c). The ^14^C-based Rubisco assays confirmed the carbon-fixation activities of heterologously expressed Rubisco and carboxysomes in *E. coli* (Fig. S4a). The overall carbon assimilation assays, performed using whole cell cultures without RuBP addition, showed that the strain harbouring the CBB cycle had a higher carbon-assimilation rate (Fig. S4b). Note that measurable ^14^C assimilation was also detected in strains without the CBB cycle, likely due to bicarbonate participation in native carbon metabolism that exchange with ^12^C. These results confirm the functionality of the heterologously expressed enzymes and highlight the significance of carboxysomes as part of the CCM in enhancing CO_2_ fixation.

To build a functional carboxysome-based CCM, we expressed DabA2/B2 in BL:Cp, along with DabA1/B1, BicA, and SbtA as alternative Ci pumps (Fig. 2b). Growth analysis revealed consistently superior performance across all carboxysome/Prk-expressing derivatives (including variants BL:CpVe, BL:CpT1, BLCpT2, BL:CpTb, and BL:CpTs) compared to those lacking these two components (BL:Veb, BL:VbT1, BLVbT2, BL:VbTb, and BL:VbTs) (Fig. 2b and d). This growth advantage is attributed to the installation of the CBB cycle, which assimilates CO₂ and thereby supplies additional carbon to promote cell growth. Notably, no significant difference in growth was detected among strains expressing DabA2/B2, DabA1/B1, BicA or SbtA alongside α-carboxysomes and Prk (Fig. 2d), despite their different Ci-importing activities determined in the Δ*C* mutant (Fig. 1d). The results suggest that additional Ci-transport capacity cannot further enhance the engineered CBB cycle in *E. coli* under these conditions, potentially due to substrate saturation or competing metabolic limitations.

### Metabolic landscape of the *E. coli* strains possessing a functional CBB cycle

To gain an understanding of how these engineering modification affects the metabolic landscape of *E. coli*, we performed metabolic profiling on the engineered *E. coli* strains expressing different combinations of metabolic modules using GC-MS (Table S1). GC-MS was chosen as methoxymation and silylation derivatisation enable the investigation of central carbon and nitrogen metabolism, in addition to other metabolic pathways and networks. This approach enabled the detection of 257 metabolic features (Supplementary File 1) and the identification of 55 metabolites (Table S2): 12 to Level 1 and 43 to Level 2 of the Metabolomics Standards Initiative [38].

**Fig. 3:**
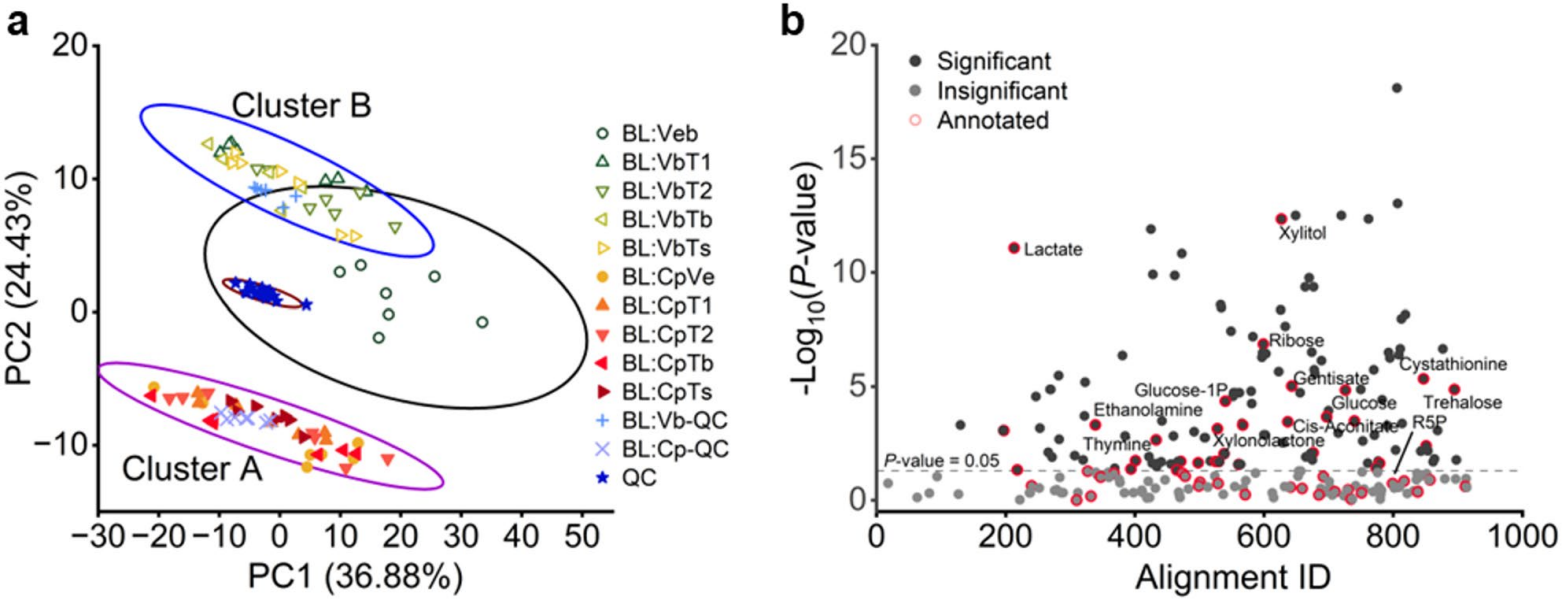
PCA and ANOVA illustrate the variances in response to the introduced components of the CBB cycle and the carboxysome-based CCM. (**a**) PCA score plot with all ten variants demonstrates a clear separation between variants harbouring the CBB cycle and those that lack it. The percentage numbers in the axis legends indicate the total variance explained by that PC. Ellipses show the 95% confidence. (**b**) ANOVA identified metabolites (or features) that were changed in response to the introduced components for the CBB cycle and CCM, FDR-corrected *P*-value threshold < 0.05

Principal Component Analysis (PCA) score plots revealed discrete clustering distributions of the engineered strains, indicating that the expression and functions of engineered proteins/assemblies resulted in metabolic changes and fundamentally restructures *E. coli* host metabolism (Fig. 3a). The distance between clusters represents the degree of their metabolic variance. Cluster A contains the strains co-expressing α-carboxysomes and Prk, along with Ci pumps (including BL:CpVe, BL:CpT1, BL:CpT2, BL:CpTs, and BL:CpTb), which are distinct from the variants expressing only Ci pumps without α-carboxysomes and Prk (BL:VbT1, BL:VbT2, BL:VbTb, and BL:VbTs), which formed Cluster B. The BL:Veb variant without expression of any recombinant proteins, was distributed separately from both Clusters A and B (Figs. 2b and 3a), confirming that expression of these modules altered the metabolic landscape significantly. Cluster A exhibited maximal separation from BL:Veb, indicating that the expression of α-carboxysomes and Prk resulted in profound metabolic changes. In contrast, Cluster B exhibited remarkably closer proximity to BL:Veb than to the BL:CpVe strain expressing only α-carboxysomes and Prk, revealing that the engineered Ci pumps alone induced comparatively modest perturbations. This hierarchy aligns with functional priorities: the CBB cycle is directly involved in the inherent PPP for carbon metabolism, whereas Ci pumps improve bicarbonate availability in the cytosol to enhance carboxylation kinetics and pH homeostasis without redirecting central carbon flux. Quantification of the total protein concentration across the engineered strains shows that total protein concentration was comparable between strains expressing various heterologous modules (Fig. S5, *p* > 0.1), excluding total protein abundance as a primary determinant of the observed metabolic changes. However, other potential metabolic consequences of heterologous protein expression, such as proteome reallocation, changes in cellular cofactor pools (NAD⁺/NADH), and resource competition for translation machinery, may still contribute to metabolic landscape remodelling and warrant further investigation in future studies.

As the pronounced metabolic shifts induced by the functional incorporation of both α-carboxysomes and Prk could potentially mask subtler differences among strains expressing various Ci pumps within the same PCA clusters (Fig. 3a), we subdivided our experimental samples to resolve these effects better. Specifically, we analyzed two separate groups: one comprising all strains harboring both α-carboxysomes and Prk (Cluster A), and another including strains lacking α-carboxysomes and Prk (Cluster B and BL:Veb). We then applied PCA and principal component-discriminant function analysis (PC-DFA) to each group independently (Figs. S6, S7). In PC-DFA, one uses an *a priori* class structure, where the aim of PC-DFA is to minimise the variance within a group and maximise variation between groups. This was performed on the biological replicates, so it is semi-supervised in nature. PC-DFA was validated by projecting samples (*n* = 1 biological replicate) not used to calibrate the model into the resulting PC-DFA ordination plot from the training data (*n* = 6 biological replicates). Our results showed no significant differences in metabolic profiles among strains expressing different Ci pumps, consistent with the findings on growth profiles of *E. coli* expressing various Ci pumps (Fig. 2d) and PCA score plots for all the strains (Fig. 3a). This suggests a largely similar metabolic influence of the engineered Ci pumps under these conditions. The lack of differentiation may reflect the limited bicarbonate requirement in wild-type *E. coli*, where endogenous CA activity may be sufficient to provide bicarbonate for cellular needs [39]. Although engineered Ci pumps can facilitate Ci import and elevate the cytosolic bicarbonate levels, excess bicarbonate may suppress their own activities [40]. In contrast, the Δ*C* mutant without endogenous CA exhibited high bicarbonate demand, maximizing the activities of Ci pumps and thus unmasking functional differences among Ci pumps (Fig. 1d). These findings highlight the role of endogenous CA in modulating the functionality of the installed CBB cycle in *E. coli*.

### Enhanced sugar accumulation in the *E. coli* host induced by the CBB cycle

**Fig. 4:**
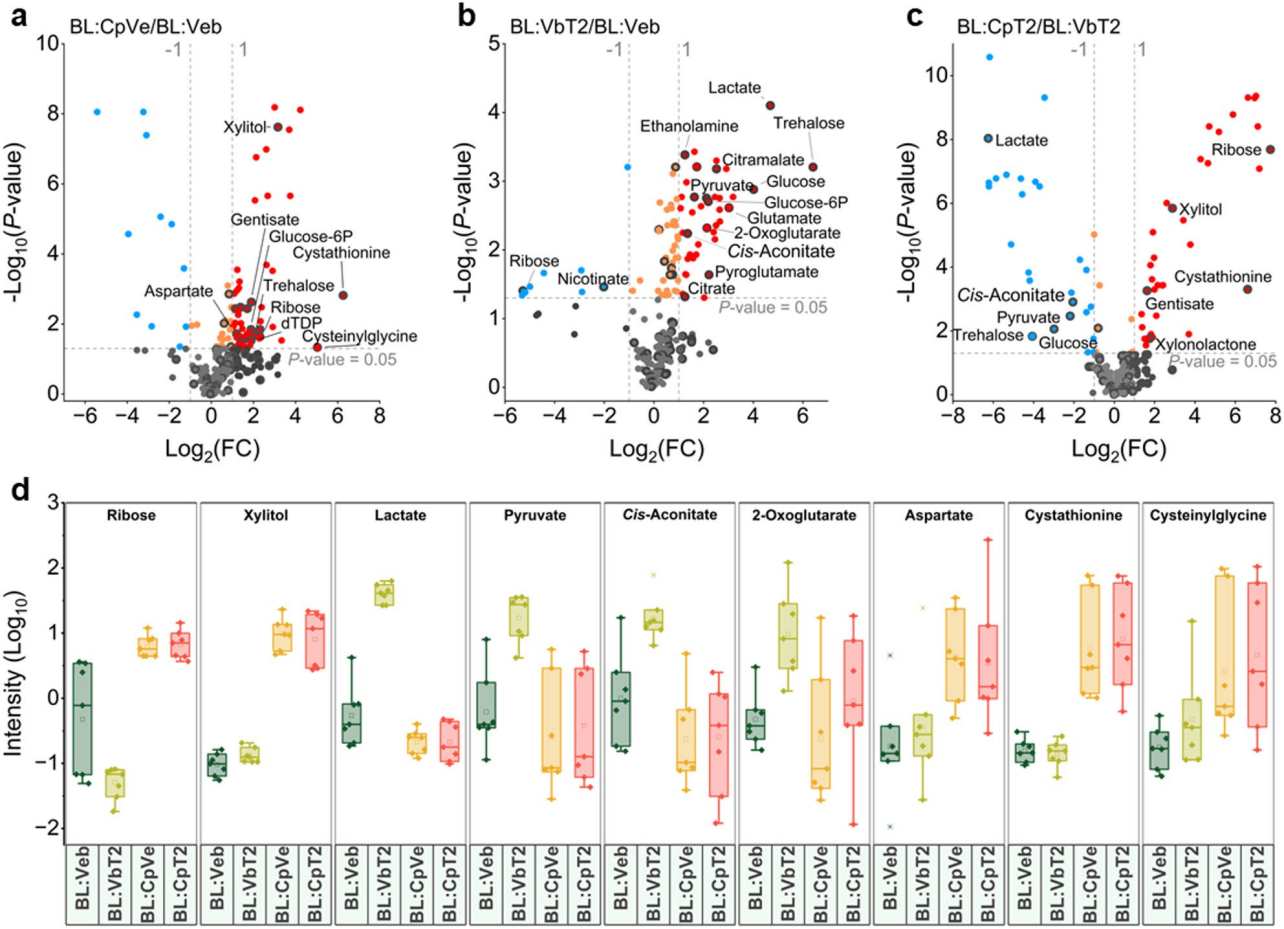
Identification of differential metabolites in response to the presence of the CBB cycle and DabA2/B2**.** Volcano plots of the paired variants, including (**a**) BLCpVe vs. BL:Veb, (**b**) BLVbT2 vs. BL:Veb, and (**c**) BL:CpT2 vs. BL:VbT2. The annotated metabolites were black cycled; blue dots, decreased (*P*-value < 0.05, Log_2_(FC) < -1); red dots, increased (*P*-value < 0.05, Log_2_(FC) > 1); yellow dots, non-significantly changed (*P*-value < 0.05, -1 < Log_2_(FC) < 1); Black and grey dots, non-significantly changed, (*P*-value > 0.05). (**d**) Boxplots represent the relative concentration of some annotated metabolites in the variants. The box range gives the interquartile range (IQR: 25% and 75%) of the data set; bars show the data within the range of 1.5 x IQR, and those data excessed this range were defined as outliers (stars). The line within the box indicates the median and the empty square (‘□’) represents the mean value

**Fig. 5:**
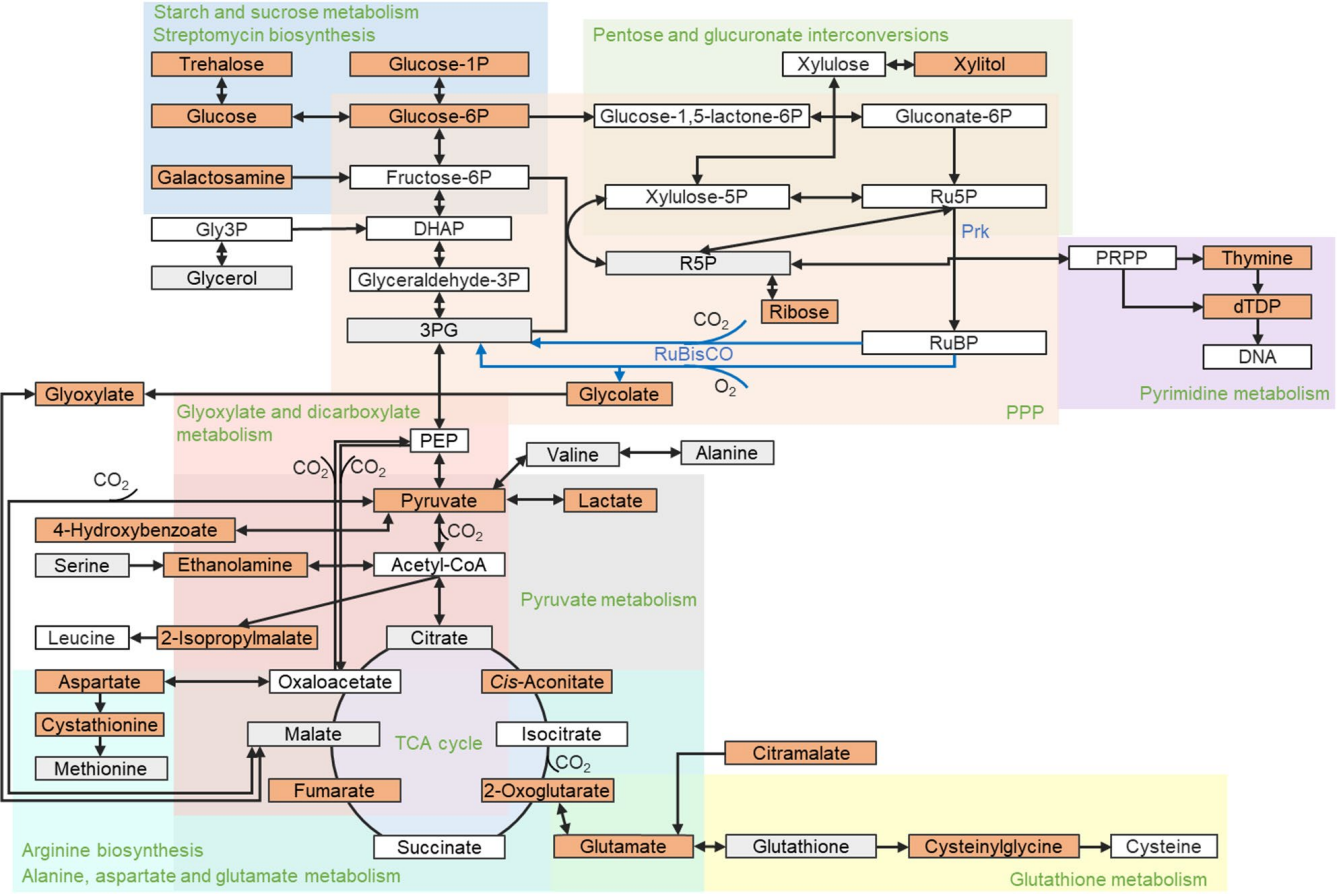
Pathways map adapted from KEGG metabolic pathways (ebl01100) of *E. coli* BL21(DE3) illustrates the connections between the affected metabolites and pathways. According to ANOVA results, metabolites that were significantly changed between the variants are shown in orange boxes, and those that were not significantly changed are in grey boxes. The white boxes give the metabolites that were not annotated from the GC-MS result. The approximate region of some pathways was highlighted with coloured shades, and the name of the pathway was displayed in green text. The two introduced enzymes, Rubisco and Prk, on the map, were given in blue. PRPP: 5-Phosphoribosyl diphosphate; PEP, Phosphoenolpyruvate; Gly3P, glycerol-3-phosphate; DHAP, dihydroxyacetone phosphate; Phosphate is abbreviated as ‘P’ in the metabolites, like fructose-6P represents fructose 6-phosphate

Since no substantial differences in growth and metabolic profiles were determined among strains expressing various Ci pumps, we focused on DabA2/B2 as the representative Ci pump for subsequent analysis of the corresponding variants (BL:Veb, BL:VbT2, BL:CpVe, BL:CpT2). ANOVA analysis revealed that 134 out of all 257 detected metabolite features showed statistically significant (false discovery rate (FDR) corrected *P*-value threshold < 0.05) differences among the engineered *E. coli* variants (Fig. 3b, Table S3). From these features, we annotated 30 metabolites (Table S4). To identify and quantify the metabolites that were specifically affected by the introduction of α-carboxysomes, Prk, and DabA2/B2, we performed pairwise comparisons of the variants with the 257 features using t-tests coupled with fold change (FC) analysis. The statistical significance was determined using FDR corrected *P*-value with threshold of < 0.05, and Log_2_(FC) > 1 (increased) or < -1 (reduced). Comparing with the negative control with only empty plasmids (BL:Veb), fourteen metabolites were increased in the presence of both α-carboxysomes and Prk, including ribose (4.94-fold), xylitol (8.94-fold), specific amino acids (e.g. aspartate: 2.30-fold, and cystathionine: 76.55-fold), and a dipeptide, cysteinylglycine (32.85-fold) (Fig. 4a and d, Fig. S8, Table S4). Integration of the CBB cycle provides an additional carbon assimilation route via the PPP, channelling Ru5P to 3PG through RuBP and Rubisco-mediated CO_2_ fixation (Fig. 5). These metabolic changes were consistent with the enhanced growth determined for strains expressing α-carboxysomes and Prk (Fig. 2c), suggesting an accelerated carbon flux.

In the global metabolic pathways of *E. coli* (Fig. 5), ribose and xylitol are synthesised from ribose 5-phosphate (R5P) and xylulose 5-phosphate (xylulose-5P), respectively. These compounds serve as precursors for Ru5P generation, which is subsequently converted to RuBP for carbon fixation via Rubisco. The increase of these two five-carbon sugars might be attributed to the coordination of the CBB cycle and the PPP, and a rerouting of carbon flux between the two pathways (Fig. 4a), which could potentially maintain R5P homeostasis (Fig. 3b) and modulating carbon fixation by tuning substrate availability [26]. Additionally, the activity of carboxysome-encapsulated CA is regulated by RuBP [41] which may suppress the conversion of bicarbonate to CO_2_, and eventually modulate the efficacy of Ci pumps in facilitating carbon fixation and cell growth (Fig. 2c).

Our analysis revealed that expression of DabA2/B2 alone led to increased levels of 14 metabolites (including lactate, pyruvate, *cis*-aconitate, 2-oxoglutarate) and a decrease of ribose (0.03-fold) and nicotinate (0.25-fold) (Fig. 4b and d, Fig. S8, Table S4). Accumulation of lactate (25.79-fold) suggests ongoing fermentation processes and excess NADH in *E. coli* [42], as lactate production serves to reoxidize NADH during glycolysis [43]. We assume that the accumulation of *cis*-aconitate (2.56-fold) and 2-oxoglutarate (4.39-fold) results from feedback inhibition in the tricarboxylic acid (TCA) cycle, where high NADH levels inhibit their conversion to fumarate, a process that also generates NADH and CO_2_ (Fig. 5). This may account for the increased levels of *cis*-aconitate and 2-oxoglutarate observed in strains expressing DabA2/B2 alone. Possibly, functional DabA2/B2 increases the cytosolic bicarbonate pool and intracellular CO_2_ levels, creating a catalytically favourable environment to stimulate fermentative metabolism.

Notably, the incorporation of the CBB cycle into DabA2/B2-expressing strains led to a substantial reduction of metabolites that were elevated with DabA2/B2 alone (Fig. 4c and d; Table S4). The lactate content was reduced (0.01-fold) in strains expressing α-carboxysomes and Prk (Fig. 4c, d), consistent with enhanced NADH consumption by the Rubisco-catalysed CBB cycle [44]. The NADH consumption may also alleviate feedback inhibition, resulting in lower levels of *cis*-aconitate (0.24-fold) (Fig. 4c, d; Table S4). Consistently, a decrease in 2-oxoglutarate (Fig. 4d) and an increase in fumarate (Fig. S8) were observed, indicating a broader metabolic shift resulted from the engineered CBB cycle. The strain expressing only Dab2 (BL:VbT2) exhibited higher glycerol consumption than the one with only empty plasmids (Fig. S9). This might also be attributed to the elevated lactate level in BL:VbT2, as less carbon was directed to biomass production (Fig. 4b). In strains containing the CBB cycle, glycerol consumption decreased, consistent with the reduced lactate accumulation observed in those strains.

In summary, installation of the CBB cycle and carboxysome-based CCM restructured the metabolic landscape, leading to the accumulation of several sugars and intermediates that could complement the enhanced growth profile associated with the CBB cycle. The increased abundance of ribose and xylitol suggests altered carbon flux exiting the PPP, highlighting a potential strategy to direct carbon flux through the engineered CBB cycle by targeted suppression of the enzymes converting xylulose-5P and R5P to xylitol and ribose, respectively.

### Network-level metabolic shifts induced by the CBB cycle and carboxysome-based CCM

**Fig. 6:**
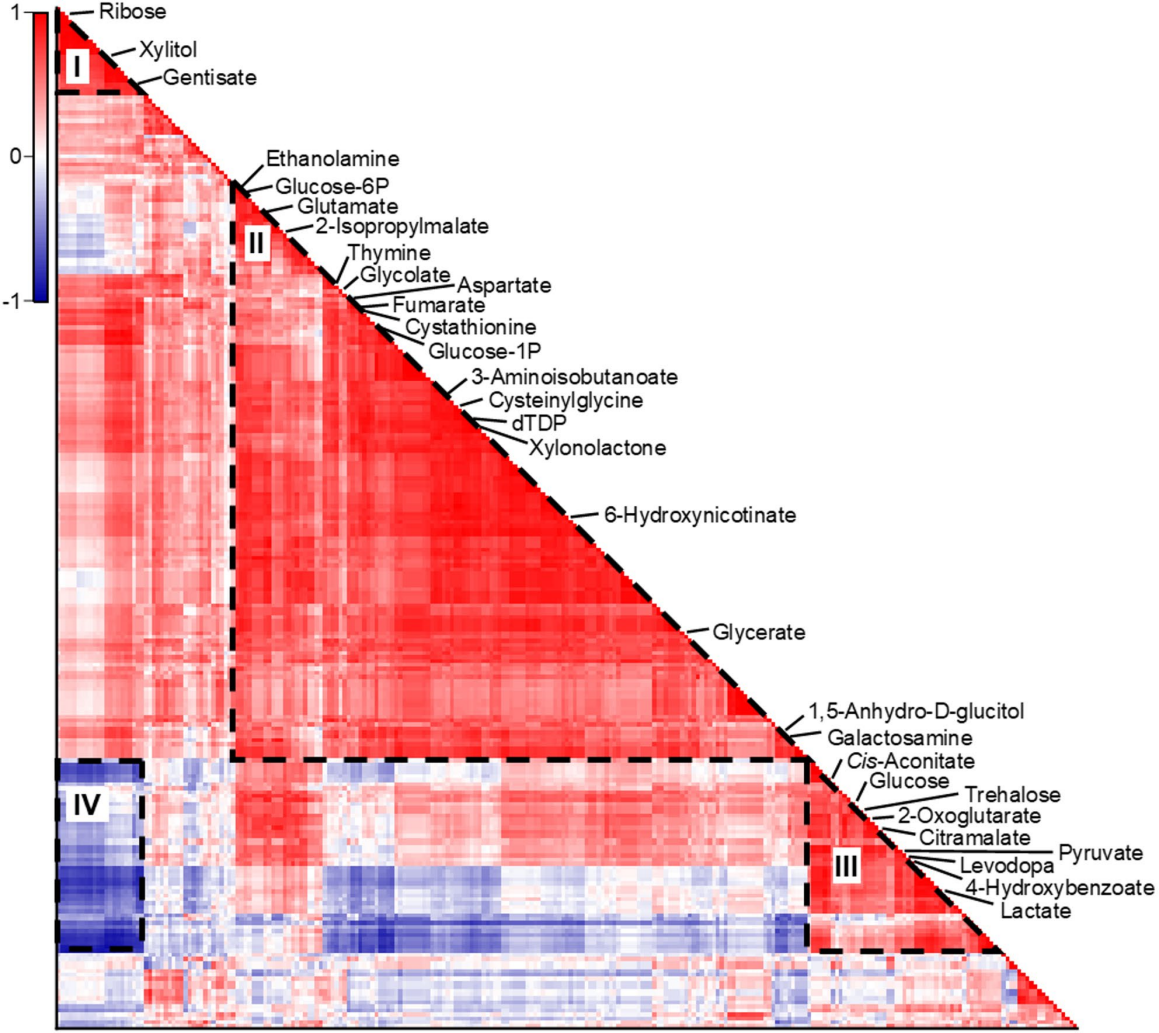
Heatmap of the correlation analysis of all 257 metabolite features**.** The x- and y-axes indicate all 257 metabolites (features), and the colour of the x-y cross indicates the Pearson’s correlation coefficient of two metabolites (-1 to 1). Four correlation clusters, marked as ‘I’, ‘II’, ‘III’, and ‘IV’, are revealed. Only labels of the 30 annotated and influenced metabolites were shown on the heatmap. The four correlation clusters were given by the dash triangles or square. Pearson’s correlation was employed for the analysis. See also Supplementary File 1

Interestingly, in addition to the 55 annotated metabolites, several features among the 202 detected metabolites also exhibited variations in response to the engineering modifications. To expand the understanding of the metabolic impact of introducing the CBB cycle alongside a carboxysome-based CCM, we performed correlation analysis on all 257 metabolites (Supplementary File 1) and identified four distinct correlation clusters (Fig. 6). Cluster I includes 22 highly positively correlated features, including ribose, xylitol, and gentisate, which were increased in the presence of the carboxysome and Prk (Fig. 4a). Cluster II, the largest group, comprises 153 positively correlated features such as ethanolamine, aspartate, glucose-6-phosphate (glucose-6P), deoxythymidine 5’-diphosphate (dTDP), and 14 other annotated metabolites that were significantly changed by the incorporation of the Prk, carboxysome and DabA2/B2. Among them, 10 were remarkably elevated in response to the installation of the CBB cycle (Figs. 4a and 6). Cluster III contains 49 metabolites which were positively correlated. Seven of them, including lactate, pyruvate, and *cis*-aconitate, were significantly increased in the strain expressing only DabA2/B2 (Fig. 4b). Cluster IV showed a negative correlation of the metabolites in Cluster I and Cluster III. This is in line with the discussion above, where the incorporation of the CBB cycle relieves the fermentation processes by consuming cytosolic CO_2_ and NADH.

Pathway analysis using the 30 alternated metabolites also revealed an extended impact on 47 metabolic pathways (Fig. S10, Table S5). Among these, six pathways in the host showed significant alterations (FDR corrected *P*-value < 0.05), including: (1) alanine, aspartate and glutamate metabolism (2), arginine biosynthesis (3), TCA cycle (4), glyoxylate and dicarboxylate metabolism (5), starch and sucrose metabolism, and (6) pyruvate metabolism.

Collectively, both metabolic profiling and correlation analysis demonstrate that incorporating a functional CBB cycle with a carboxysome-based CCM into *E. coli* reprogrammed the metabolic landscape of the host cells. Expansion of our in-house metabolite reference library would further strengthen identification confidence, enhance annotation of presently uncharacterized metabolic features and potentially reveal additional affected metabolites and pathways in future metabolomic analysis, and thereby, enriching our understanding of the system-wide metabolic rewiring induced by the engineered systems. Apart from metabolomic analysis, future studies should integrate transcriptomics, proteomics, enzymatic assays, and quantitative flux analyses to better understand how foreign pathways affect metabolic regulation, and test inactive gene variants to differentiate the effects of protein function and protein expression burden. Additionally, limitations like substrate saturation and metabolic bottlenecks restrict further improvements in carbon fixation, emphasizing the need for target metabolic and enzyme engineering.

## Conclusions

In this work, we engineered a complete, functional CBB cycle in *E. coli*, by incorporating α-carboxysomes and Ci pumps derived from *H. neapolitanus*, as well as cyanobacterial Prk, to enhance carbon assimilation in heterotrophic organisms. Among the four tested Ci pumps, DabA2/B2 exhibited the highest Ci-import activity. The installation of α-carboxysomes improved carbon fixation in the engineered strains. Comprehensive metabolic profiling revealed distinct metabolic adaptations in response to the engineered CBB cycle and CCM system. This allowed us to identify the accumulation of xylitol and ribose as potential metabolic bottlenecks amenable to future engineering. While a deeper understanding of these metabolic shifts is essential for elucidating the mechanistic basis for the observed phenotypes, our findings provide valuable guidance for the rational optimization of artificial carbon-fixation modules using synthetic biology and lay the framework for targeted metabolic engineering to be leveraged for diverse biotechnological applications, such as carbon capture and storage, crop improvement, and the production of biofuels and green chemicals.

## Materials and methods

### Bacteria and cultivation conditions

The *E. coli* strain BL21(DE3) was utilised as a platform for expressing the recombinant proteins and integrating the CBB cycle. Cultures were grown aerobically in lysogeny broth (LB) medium (10 g L^− 1^ tryptone (Apollo Scientific, BIT1332), 5 g L^− 1^ yeast extract (Millipore, 1.03753), and 10 g L^− 1^ NaCl (Fisher Scientific, 10735921)) or on LB agar (LB with 1.5% (w:v) agar (Fisher Scientific, 10548030)) plates. The high CO_2_-dependent mutant strain was maintained under 10% CO_2_ conditions in a CO_2_ incubator (MCO-18AC, PHCbi, USA). Antibiotics were added to the culture medium or agar plates at final concentrations of 50 µg mL^− 1^ chloramphenicol (Sigma-Aldrich, C0378) and 50 µg mL^− 1^ kanamycin (Melford, K22000), as required. Protein expression was induced with appropriate inducers at final concentration of 0.5 mM isopropyl β-D-1-thiogalactopyranoside (IPTG; Melford, 2946-I56000) or 1 mM arabinose (Melford, A51000). Minimal medium (M9; 47.8 mM Na_2_HPO_4_, 22.0 mM KH_2_PO_4_, 18.7 mM NH_4_Cl, 8.7 mM NaCl, 0.010 mM CaCl_2_, 0.100 mM MgSO_4_, 0.005 mM MnCl_2_, 0.012 mM ZnCl_2_, 0.003 mM CuCl_2_, 0.003 mM CoCl_2_, 0.002 mM Na_2_MoO_4_), containing glycerol as the sole carbon source, was supplemented with the appropriate antibiotics and inducers to illustrate the growth profile of the variants.

### Construction of expression plasmids for recombinant proteins

Genes encoding DabA1/B1 and DabA2/B2 were cloned from *H. neapolitanus* (NC_013422.1) [35], while genes encoding BicA and SbtA were cloned from *Synechocystis* sp. PCC 6803 (NC_000911.1). These genes were individually inserted into linearized vector pET28a, generating the corresponding expression plasmids (Fig. 1a). Hexahistidine tags (6xHis-tag) were attached to the recombinant proteins for validation of their expression through immunoblot analysis.

The α-carboxysome expression plasmid (pCB) (Fig. 2a) was acquired from a previous study [20]. In brief, all ten genes encoding the components of α-carboxysomes from the chemolithoautotrophic proteobacterium *H. neapolitanus* [45], namely, *cbbL*, *cbbS*, *csoS2*, *csoSCA*, *csoS4A/B*, and *csoS1A/B/C/D*, were amplified from the pHnCBS1D (Plasmid #52065, Addgene, UK), and assembled into the pBAD33 vector amplicon [46].

The *prk* gene was first cloned from *Synechococcus elongatus* PCC 7942 (GenBank: CP000100.1), and assembled into the pBAD33 backbone with a 6xHis-tag at its N-terminus. Then, it was amplified, along with the 6xHis-tag, and inserted downstream of *csoS1D* on pCB, resulting in the co-expression plasmid pCBPRK. All eleven genes, comprising ten for α-carboxysomes and one for Prk, were regulated by the *araBAD* promoter in this construct. A Rubisco and Prk co-expression plasmid (pRubPRK) was generated by removing the genes for the α-carboxysome shell components and the encapsulated CA from pCBPRK (Fig. 2a).

### Generation of the high CO_2_-requiring mutant

The endogenous CA encoding gene, *can*, was eliminated from the chromosome of BL21(DE3) using the I-*Scel* mediated genomic editing method [47, 48], generating the high CO_2_-requiring mutant for evaluating the activity of Ci pumps. Briefly, two homologous arms (~ 500 bp each) flanking the *can* gene were amplified from the wild-type BL21(DE3) and inserted into the helper plasmid pEMG between two I-*Scel* cleavage sites, creating the plasmid pEMG-Δ*can*. This plasmid was then transformed into the helper strain S17-1λ*pir* via electroporation for selection and reproduction, followed by conjugation with the target strain BL21(DE3). After recombining the helper plasmid into the chromosome of BL21(DE3), the target gene, along with the recombined helper plasmid, was cleaved from the chromosome by the I-*SceI* endonuclease, resulting in the mutant lacking the *can* gene (Δ*C*). The desired Δ*C* mutant was selected and confirmed using PCR analysis and growth profiling. The helper plasmids were later eliminated from the mutant by cultivating it in an antibiotic-free medium.

### Growth profile measurement

Growth curves were measured using a growth profiler (GP960, EnzyScreen, Netherlands). Seeding cells were harvested and washed after overnight incubation in LB medium supplemented with the appropriate antibiotics and inducers. The initial optical density at 600 nm (OD_600_) of the culture was adjusted to 0.1 absorption unit (a.u.), and 250 µL of the culture was inoculated into a 96-well plate, with 6 replicates (or 5 if specified) for each colony. The plate was incubated at 37 °C under ambient air conditions with continuous shaking at 225 revolutions per minute (rpm).

### In vivo carbon assimilation assays

The carbon fixation activity of Rubisco/carboxysome in the engineered strains was measured using a method adapted from previous studies [49–51]. Specifically, engineered strains were cultivated in M9 medium supplemented with 0.4% glycerol and appropriate antibiotics and inducers. Cells were harvested and washed thrice with assay buffer (100 mM HEPPS, pH 8.0, and 20 mM MgCl_2_), then resuspended in assay buffer to an OD_600_ of 4 a.u. The cell suspension was mixed with an equal volume of assay buffer containing 0.12% (w/v) alkyltrimethylammonium bromide and 48 mM NaH^14^CO_3_, and incubated at 37 °C for 2 min. The reaction was initiated by adding RuBP (final concentration 1 mM) and terminated after 5 min with 10% formic acid. Samples were dried at 95 °C and then resuspended in distilled water. Radioactivity was measured using a scintillation counter (Tri-Carb; Perkin-Elmer) in the presence of scintillation cocktail (Ultima Gold XR; Perkin-Elmer). The ^14^C assimilation rate was calculated and normalised to OD_600_. Overall carbon assimilation by the engineered strains was assessed using a similar ^14^C-based method but without RuBP supplementation. Cell cultures were mixed with NaH^14^CO_3_ (final concentration 24 mM) and incubated at 37 °C for 30 min. The reaction was terminated by adding 10% formic acid, and the samples were processed and measured as detailed above.

### GC-MS metabolic profiling

Samples for metabolic profiling were prepared following a method adapted from a previous study [52]. Cultures of the variants, with 7 replicates each, were harvested at mid-log phase and quenched immediately by mixing 15 mL of culture with 30 mL of cold (-48 °C) aqueous methanol (methanol:water, 3:2). The cells were immediately pelleted via centrifugation at -9 °C, 4,800 *g* for 10 min, and then stored at -80 °C. Intracellular metabolites were extracted from the cell pellets using 1 mL extraction solution (methanol:water, 4:1, -48 °C) through three flash-freeze and thawing cycles. After removing cell debris via centrifugation at 21,000 *g* for 10 min at -9 °C, the supernatant was collected and normalized based on the OD_600_ measured at the time of sample collection. Pooled quality control (QC) samples were created by mixing portions from each sample [53]. All samples were spiked with a mixture of deuterated chemicals as internal standards, and the solvents were removed using a vacuum concentrator (Vacufuge Vacuum concentrator 5301, Eppendorf, USA) for a minimum of 4 h to pellet the metabolites.

The extracted metabolites were derivatized using a two-step derivatization approach adapted from Begley, Francis-McIntyre [54]. Methoxylamine hydrochloride (A0415450, Acros Organics) was dissolved in dry pyridine (20 mg mL^− 1^), and 50 µL was added to each dried metabolite pellet. The mixtures were then incubated at 65 °C for 40 min. Afterwards, 50 µL of *N*-Methyl-*N*-trimethylsilyltrifluoroacetamide (MSTFA; TS-48913, Thermo Scientific, USA) was added to each sample, followed by another 40 min incubation at 65 °C. A solution containing five alkane chemicals, used as retention indexes, was spiked into each sample before centrifugation at 13,500 *g* for 15 min [55]. Subsequently, 100 µL of the supernatant, avoiding any solid residues, was transferred to GC vials (5190–9590, Agilent, USA) and ready for GC-MS analysis.

GC-MS analysis was carried out using an Agilent GC-MS system (8890 GC system tended with 7250 GC-qToF-MS, Agilent, USA) and an HP-5ms (30 m, 0.25 mm, 0.25 μm) capillary column (Agilent, USA), following methods adapted from a previous study (Dunn et al., 2011). One microliter of the sample was injected into the inlet (280 °C) and processed to the column under a split ratio of 20:1. Helium gas was used as the carrier gas, maintained at a constant flow rate of 1 mL min^− 1^. The oven temperature was initially set to 70 °C and held for 4 min, then ramped to 300 °C at a rate of 20 °C per minute and held for an additional 4 min. Following each injection, the oven temperature was reduced from 300 °C to 70 °C in preparation for the next sample. The mass spectrometer operated in electron ionisation (EI) mode with an electron energy of 70 eV. The detector acquired signals across a *m*/*z* range of 45–600 at a rate of 20 Hz. The raw data was converted to ‘*.mzML’ format using the MSConvert [56], and then deconvoluted using MS-DIAL [57]. The Golm Metabolome Database (GMD) [58] and a combined database from the MS-DIAL project (Library with all records using Kovats retention index) [55, 57, 59] were employed to annotate the metabolic results. Standard chemicals were also utilised to provide a level I annotation for some metabolites, following the Metabolomics Standards Initiative Guidelines [38].

### Statistical analysis

The peak intensity of the metabolite features was logarithmically (log_10_) transformed and then autoscaled, where each feature is first mean-centred and then divided by its standard deviation. Principal component analysis (PCA) and principal component-discriminant function analysis (PC-DFA) [60–62] were performed on this pre-processed dataset using MATLAB (MatLabR2023b, MathWorks). Analysis of Variance (ANOVA) and correlation analysis were conducted using MetaboAnalyst [63, 64]. In order to compensate for multiple testing and reduce the FDR, Benjamini-Hochburg procedure [65] adjusted *P*-values with threshold of < 0.05 were used to determine the statistical significance. Pearson correlation analysis was employed to assess the distance in correlation patterns among metabolites.

### Glycerol consumption assay

Cells were cultivated in M9 medium containing 0.4% glycerol along with appropriate antibiotics and inducers. Cultures were harvested at two time points within the exponential phase at equal time intervals. After spinning the cells down, the supernatants were collected, and the glycerol concentrations were measured using a Glycerol Assay Kit (Merck, MAK117). Glycerol consumption between the two sampling points was then normalised to the increase in OD_600_ over the same period.

### Pathway analysis

Pathway analysis was carried out in MetaboAnalyst [63, 64], with *E. coli* K-12 MG1655 (KEGG) as the reference organism. To visualise the connections between the identified metabolites, a pathway map was generated based on the metabolic pathways of BL21(DE3) in KEGG [66]. The streptomycin biosynthesis pathway was removed from the result since only a few shared reactions can be fulfilled in *E. coli* BL21(DE3), though it was identified as significantly affected.

### Protein concentration quantification

Cells were cultivated in M9 medium supplemented with 0.4% glycerol and appropriate antibiotics and inducers. They were harvested, washed thrice with buffer (100 mM HEPPS, pH 8.0, and 20 mM MgCl_2_), and resuspended in the same buffer to an OD_600_ of 4.0 a.u. Cells were lysed using a probe sonicator for 6 rounds of 3 s on and 7 s off. Total protein concentration was determined using the Bradford assay [67, 68]. Differences between samples were evaluated by one-way ANOVA, followed by means comparison using the Tukey Test.

## Supplementary Information

Below is the link to the electronic supplementary material.

Supplementary Material 1

Supplementary Material 2

## Data Availability

Data is provided within the manuscript or supplementary information files.

## Abbreviations

CO_2_: Carbon dioxide
CBB cycle: Calvin-Benson-Bassham cycle
GC-MS: Gas chromatography-mass spectrometry
Rubisco: Ribulose 1,5-bisphosphate carboxylase/oxygenase
Ci: Inorganic carbon
CCM: CO_2_-concentrating mechanism
CA: Carbonic anhydrase
PPP: Pentose phosphate pathway
Prk: Phosphoribulokinase
Ru5P: Ribulose-5-phosphate
RuBP: Ribulose-1,5-bisphosphate
3PG: 3-Phosphoglycerate
6xHis-tags: Hexahistidine tags
SDS-PAGE: Sodium dodecyl sulfate-polyacrylamide gel electrophoresis
PCA: Principal Component Analysis
ANOVA: Analysis of variance
*p*-value: Raw *p*-value from statistical analysis
PC-DFA: Principal component-discriminant function analysis
FDR: False discovery rate
*P*-value: Raw *p*-value corrected by FDR
FC: Fold change
R5P: Ribose 5-phosphate
Xylulose-5P: Xylulose 5-phosphate
TCA: Tricarboxylic acid
Glucose-6P: Glucose-6-phosphate
dTDP: Deoxythymidine 5’-diphosphate
IPTG: Isopropyl β-D-1-thiogalactopyranoside
a.u.: Absorption unit
OD_600_: Optical density at 600 nm
rpm: Revolutions per minute
QC: Quality control
PRPP: 5-Phosphoribosyl diphosphate
PEP: Phosphoenolpyruvate
Gly3P: glycerol-3-phosphate
DHAP: dihydroxyacetone phosphate
HEPPS: 3-[4-(2-Hydroxyethyl)-1-piperazinyl]-propanesulfonic acid
