# Supplementary material for: Reprogramme the E. coli metabolism by engineering a functional carbon-fixation pathway
Source: J Biol Eng. 2025 Dec 29;20:21. doi: 10.1186/s13036-025-00612-x (PMC12859837; doi:10.1186/s13036-025-00612-x)
Supplement: Supplementary file 2 — Supplementary Material 2 [file 13036_2025_612_MOESM2_ESM.docx]

**Supplemental information**

**for**

**Reprogramme the *E. coli* metabolism by engineering a functional carbon-fixation pathway**

Yu Chen^1,2^, Adam Burke^2^, Vincent Chriscoli^1^, Mengru Yang^1^, Ping Chang^1^, Tianpei Li^1^, Buke Zhang^3^, Royston Goodacre^2^*, Lu-Ning Liu^1,3^*

^1^Department of Biochemistry, Cell and Systems Biology, Institute of Systems, Molecular and Integrative Biology, University of Liverpool, Liverpool L69 7ZB, United Kingdom

^2^Centre for Metabolomics Research, Department of Biochemistry, Cell and Systems Biology, Institute of Systems, Molecular and Integrative Biology, University of Liverpool, Liverpool L69 7ZB, United Kingdom

^3^MOE Key Laboratory of Evolution and Marine Biodiversity, Frontiers Science Center for Deep Ocean Multispheres and Earth System & College of Marine Life Sciences, Ocean University of China, Qingdao 266003, China

*Correspondence: [luning.liu@liverpool.ac.uk](mailto:luning.liu@liverpool.ac.uk) (L.-N.L.); [roy.goodacre@liverpool.ac.uk](mailto:roy.goodacre@liverpool.ac.uk) (R.G.)


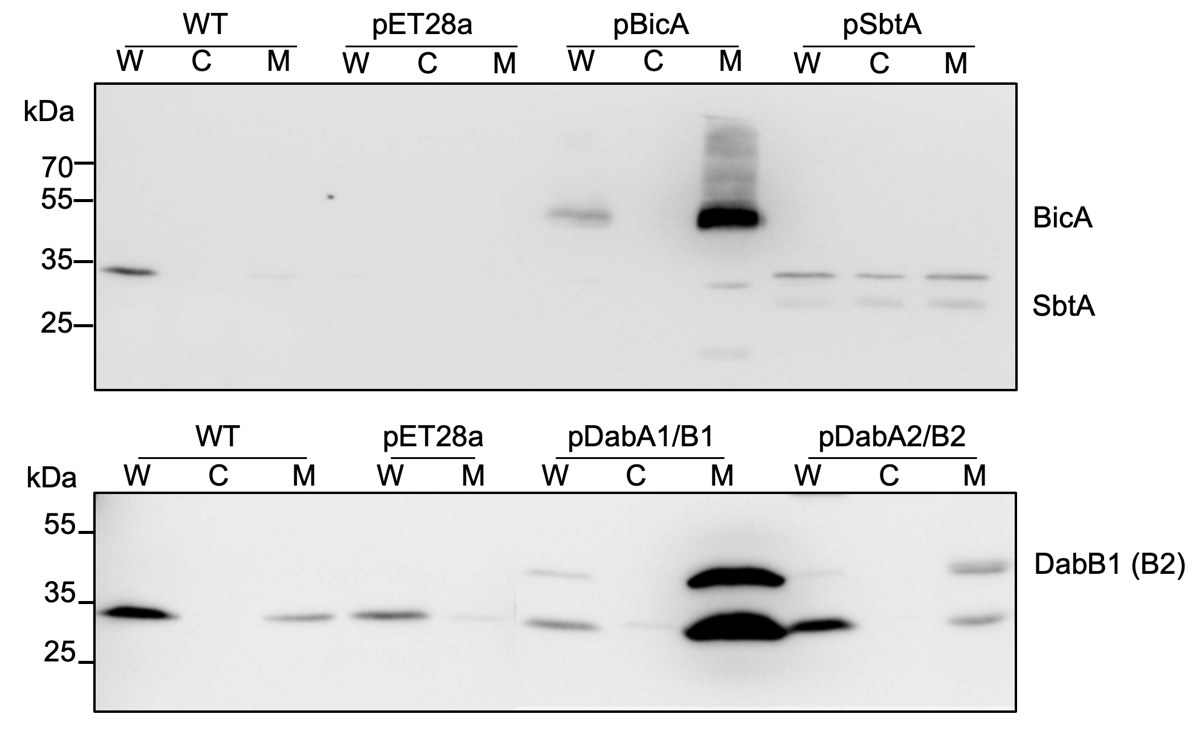


Fig. S1. Immunoblots illustrate the heterogeneously expressed Ci pumps in the membrane fractions of *E. coli*. Abbreviations: WT: wild-type BL21(DE3); W: whole-cell samples, with both cytoplasm and membrane unseparated; C: cytoplasm samples; M: isolated membrane samples. Anti-His antibody was applied for the detection. The bands at about 35 kDa are nonspecific binding of the 6xHis-tag antibody.

**
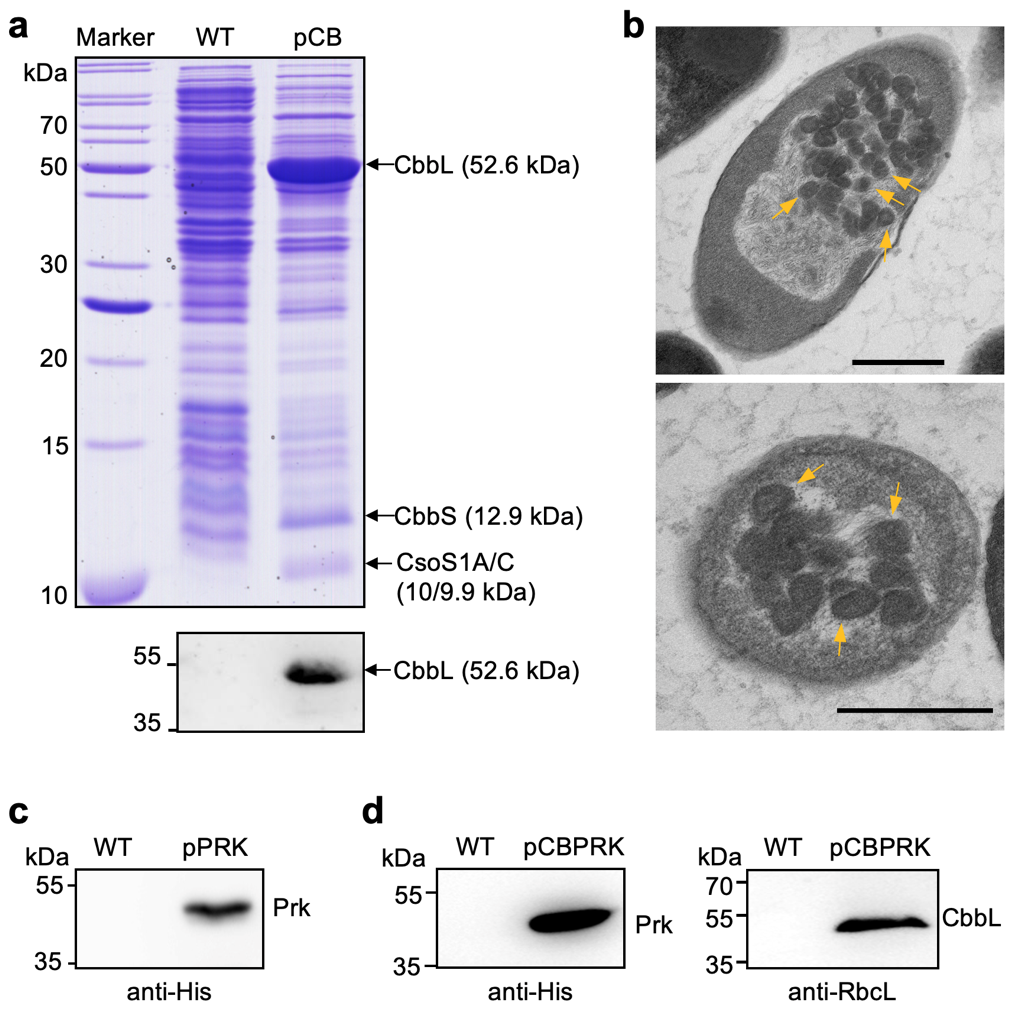
**

Fig. S2. Protein expression in *E. coli*. The expressed α-carboxysomes were detected using (a) SDS-PAGE and immunoblot analysis, and (b) thin-section electron microscopy; yellow arrows pointed out a few carboxysomes in the images; scale bar: 500 nm. (c) Prk expression was validated using immunoblot analysis. (d) The co-expression of carboxysome components and Prk was also confirmed using immunoblot analysis.


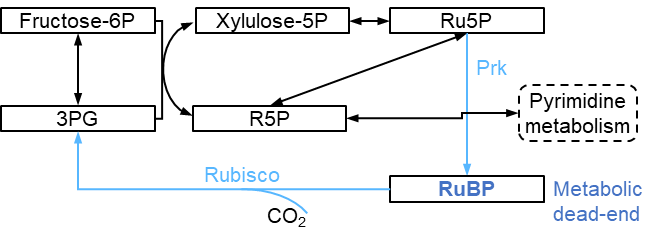
Fig. S3. Pathway map adapted from KEGG metabolic pathways of ***E. coli*** BL21(DE3) illustrates the Prk inhibitory effect. Heterologous proteins introduced into *E. coli* are shown in light blue. Ribulose 1,5-bisphosphate (RuBP) converted from ribulose 5-phosphate (Ru5P) is the metabolic dead end of the pathway when Rubisco is absent from the system. The dashed rounded rectangle gives the metabolic pathway downstream.


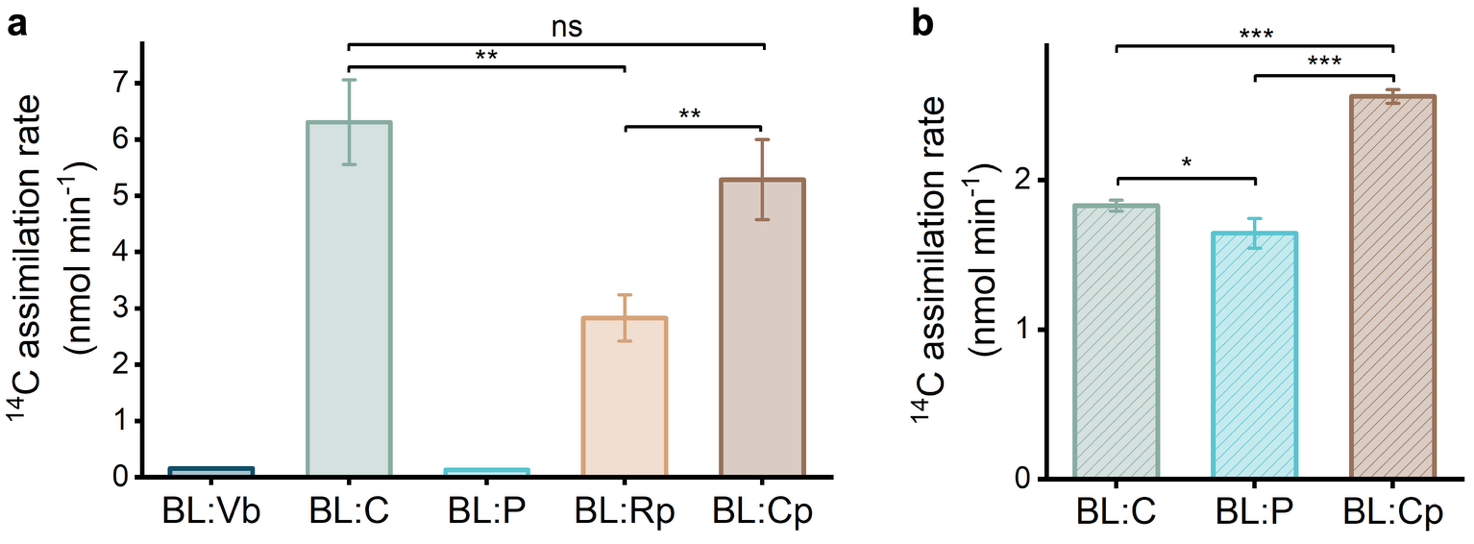


Fig. S4. Carbon assimilation rates in engineered *E. coli*. (a) Carbon-fixation activities specific to the heterologously expressed Rubisco or carboxysomes. RuBP was added to saturate Rubisco in a 5 min reaction. (b) Overall carbon-assimilation rates of variants with or without the CBB cycle. Cell cultures were used in a 30 min reaction without RuBP. ^14^C assimilation rates were normalized to OD_600_. Statistical significance was assessed using a two-tailed t-test and indicated by stars. ns: not significant (*p*-value > 0.05); *: *p*-value < 0.05; **: *p*-value < 0.01; ***: *p*-value < 0.001.


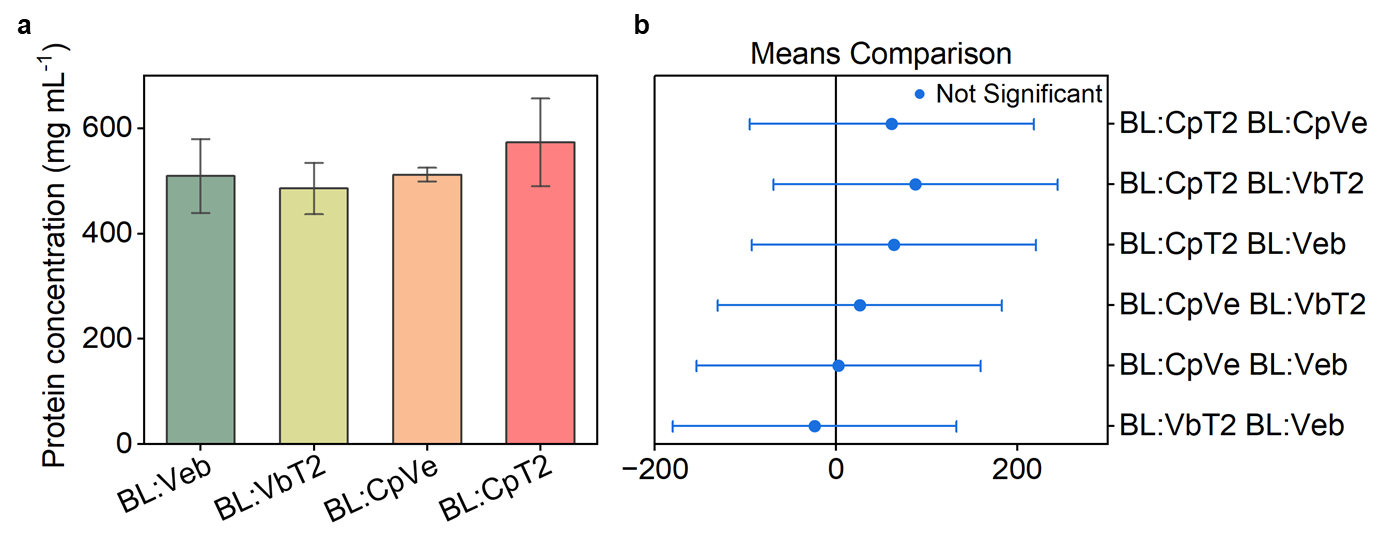


Fig. S5: Total protein concentrations and mean comparison for the engineered strains. (a) Total protein concentrations were measured from cell lysates prepared from suspensions with OD_600_ adjusted to 4 a.u. (b) Mean comparisons were carried out using the Tukey test. Blue dots closer to zero indicate smaller differences between means. Error bars represent the limits of agreement, and intersections with the zero line denote a non-significant difference.

**
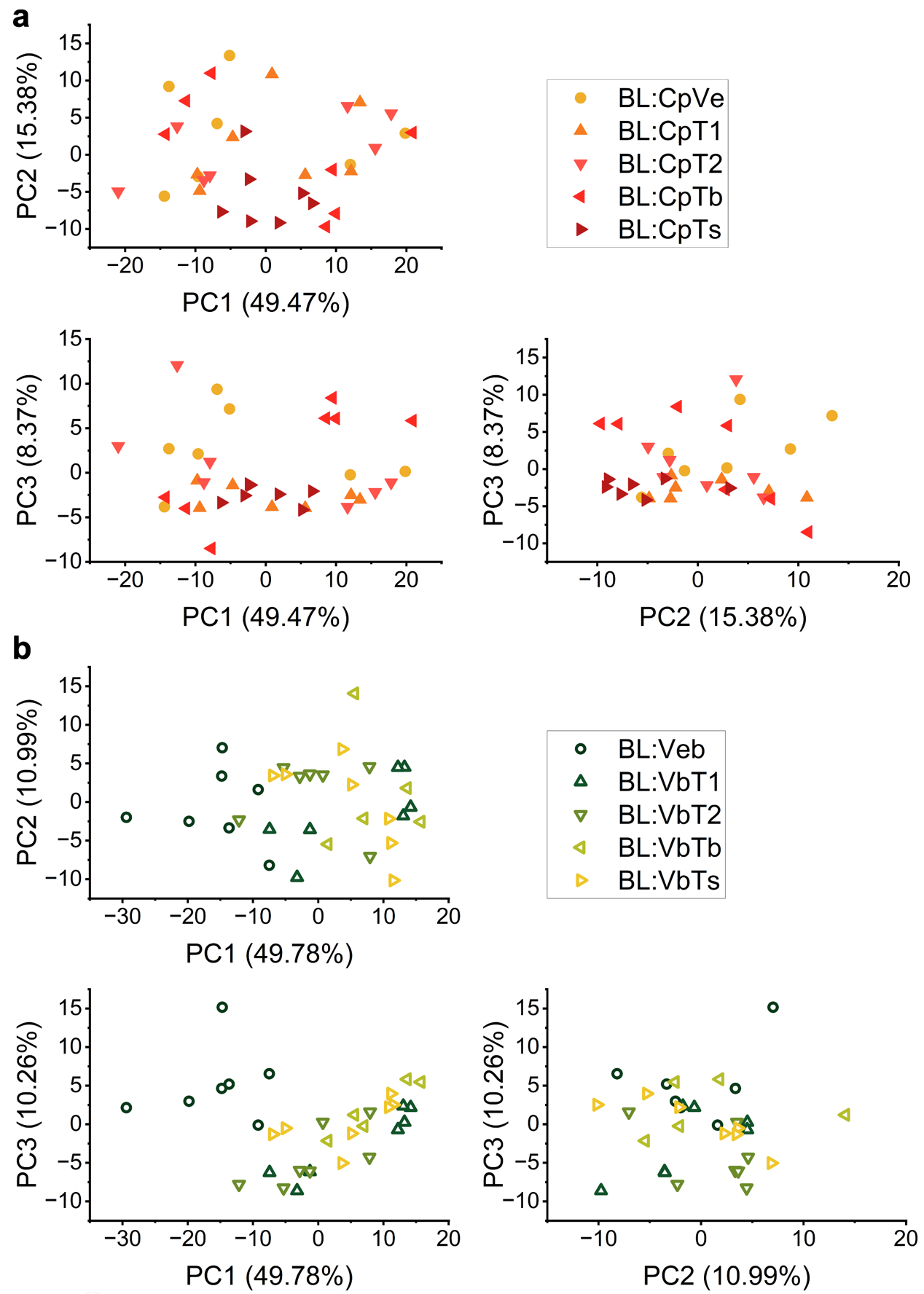
**Fig. S6: PCA score plots illustrate the variances within the two blocks of the variants. (a) Score plots of variants harbouring the CBB cycle, along with empty plasmid pET28a or Ci pumps. (b) Score plots of variants lacking the CBB cycle but possessing empty plasmid pET28a or Ci pumps. Twenty PCs were extracted with the first three being plotted here to demonstrate the distribution of samples. The percentage numbers in the axis legends indicate the total variance explained by that PC.

**
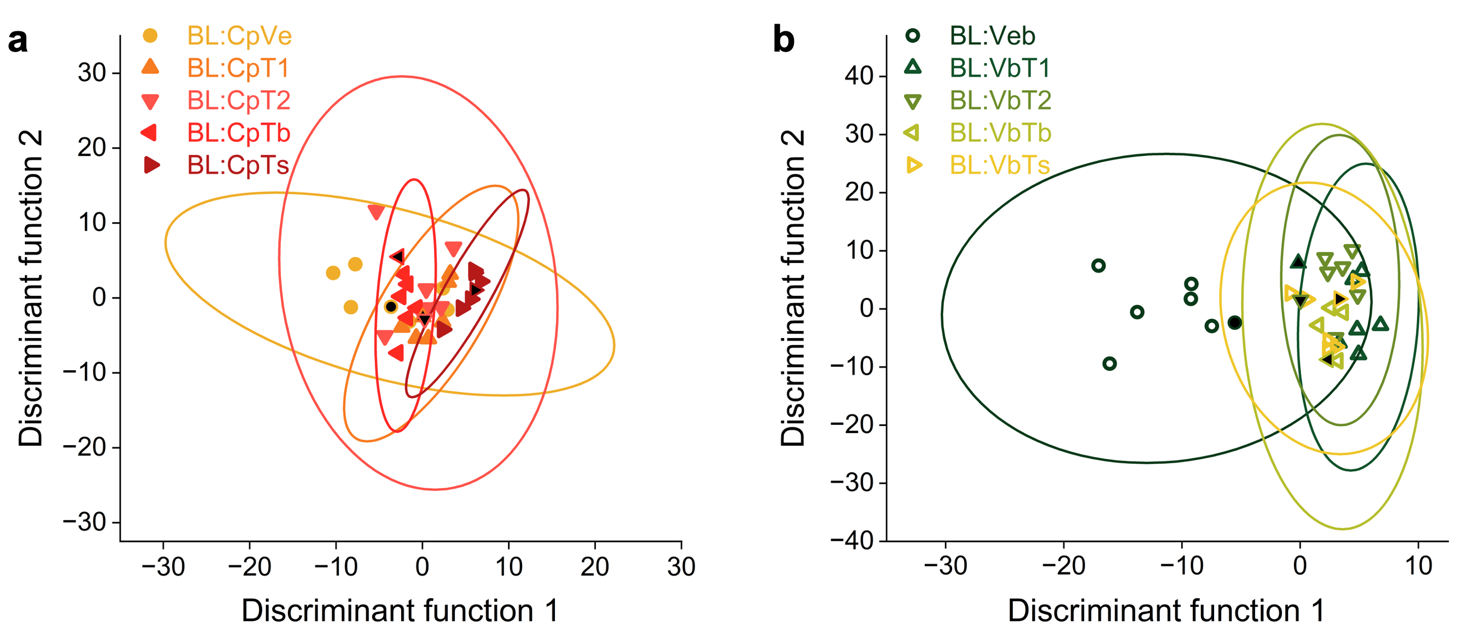
**Fig. S7. PC-DFA score plots of the two blocks of variants. (a) score plot of the strains installed with the CBB cycle, along with different Ci pumps or empty plasmid pET28a. (b) PC-DFA score plot of the strains that lack the CBB cycle while expressing different Ci pumps or possessing empty plasmid pET28a. The ellipses show the 95% confidence region for each group. The PC-DFA score plots were generated with six replicates randomly selected from the total seven replicates. The remaining replicate was used to test the PC-DFA by projecting those samples on the score plots (black-filled shapes).





Fig. S8. Boxplot of the metabolites that were influenced by the presence of the CBB cycle and carboxysome-based CCM. Each variant has seven biological replicates shown as seven data points; the box range gives the interquartile range (IQR: 25% and 75%) of the data set; bars show the data within the range of 1.5 x IQR, and data exceeding this range were defined as outliers (stars).

**
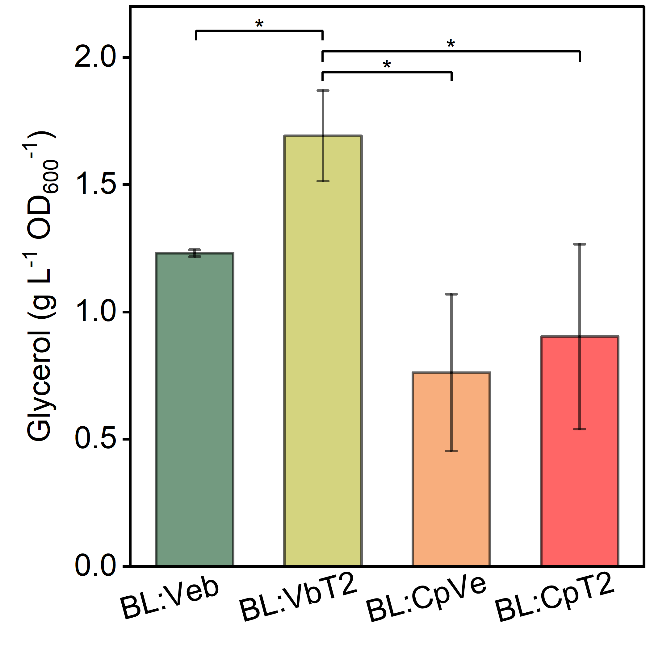
Fig. S9. Glycerol consumption in engineered *E. coli*.** The glycerol consumption during the exponential phase of the same time interval was measured and normalized to the OD_600_ increment. Means comparison was carried out using a t-test with a threshold of 0.05. ‘*’ *p*-value < 0.05.

**
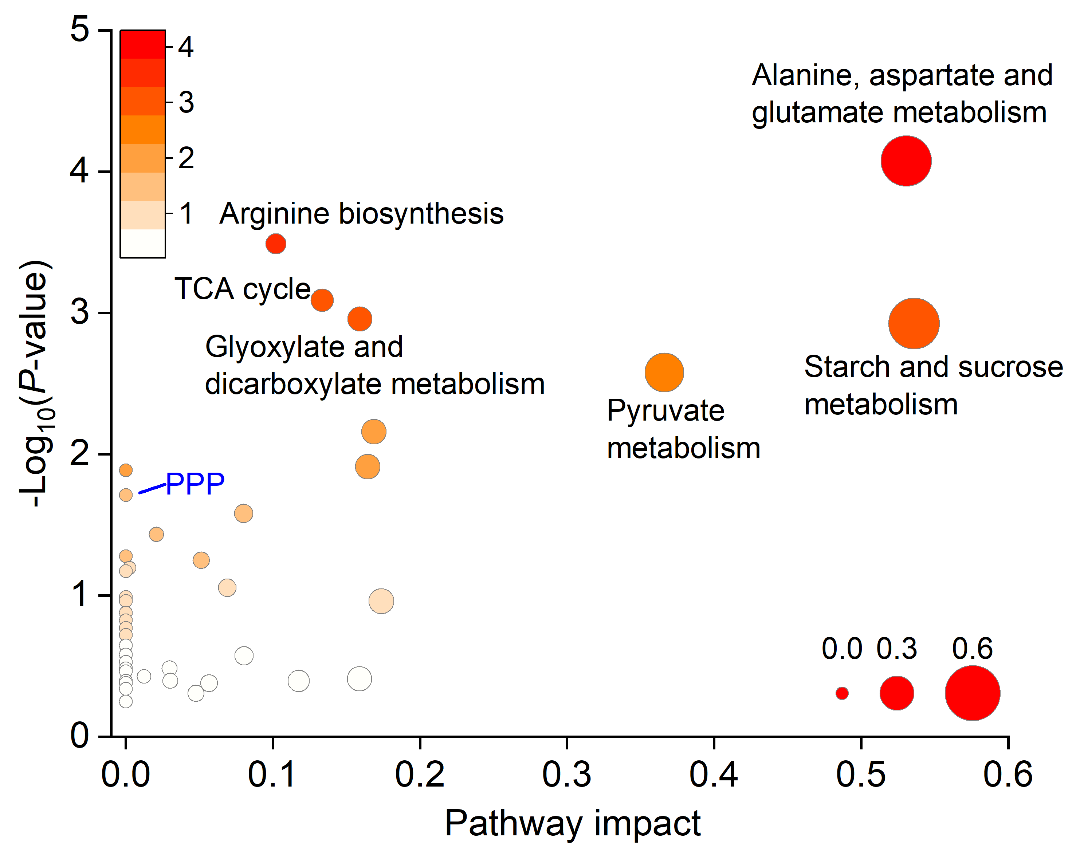
**Fig. S10. Pathway analysis result revealed 30 potential altered metabolic pathways after the installation of the CBB cycle and CCM in *E. coli*. The first 6 pathways that were significantly affected are labelled. The pentose phosphate pathway, in which the carbon-fixation pathway is involved, was also outlined (label in blue). The colour scale bar (inset) indicates the logarithmically transformed (log_10_) *P*-value (FDR corrected) depicted on Y-axis. The circle scale bar (inset) indicates the pathway impact depicted on X-axis.

Table S1. Bacterial variants used for GC-MS analysis and the corresponding subset biological QCs and pooled QCs. Subset QCs BL:Vb-QCs including aliquots from variants which lack the CBB cycle, and subset QCs BL:Cp-QCs including aliquots from variants that with the CBB cycle installed. The pooled QCs including aliquots from all variants.

| **Variants label** | **Plasmids possessed** | **Subset QCs** | **Pooled QCs** |
| --- | --- | --- | --- |
| BL:Veb | pBAD33 and pET28a | BL:Vb-QCs  7 replicates | Pooled QCs  21 replicates |
| BL:VbT1 | pBAD33 and pDabA1/B1 |  |  |
| BL:VbT2 | pBAD33 and pDabA2/B2 |  |  |
| BL:VbTb | pBAD33 and pBicA |  |  |
| BL:VbTs | pBAD33 and pSbtA |  |  |
| BL:CpVe | pCBPRK and pET28a | BL:Cp-QCs  7 replicates |  |
| BL:CpT1 | pCBPRK and pDabA1/B1 |  |  |
| BL:CpT2 | pCBPRK and pDabA2/B2 |  |  |
| BL:CpTb | pCBPRK and pBicA |  |  |
| BL:CpTs | pCBPRK and pSbtA |  |  |

Table S2. All 55 annotated metabolites with their corresponding KEGG IDs and annotation confidence levels.

| **Alignment ID** | **KEGG ID** | **Metabolite names** | **Confidence level^§^** | **Reference** |
| --- | --- | --- | --- | --- |
| 197 | C00022 | Pyruvate | 2 | MSDIAL-DB**^#^** |
| 213 | C00186 | Lactate | 2 | MSDIAL-DB |
| 218 | C00160 | Glycolate | 2 | MSDIAL-DB |
| 240 | C00041 | Alanine | 1 | STD* in this study |
| 310 | C00183 | Valine | 1 | STD in this study |
| 327 | C00013 | Pyrophosphate | 2 | MSDIAL-DB |
| 332 | C00065 | Serine | 2 | MSDIAL-DB |
| 339 | C00189 | Ethanolamine | 2 | MSDIAL-DB |
| 347 | C00116 | Glycerol | 2 | MSDIAL-DB |
| 368 | C00253 | Nicotinate | 2 | MSDIAL-DB |
| 394 | C00258 | Glycerate | 2 | MSDIAL-DB |
| 401 | C00122 | Fumarate | 2 | MSDIAL-DB |
| 433 | C00178 | Thymine | 2 | MSDIAL-DB |
| 465 | C03284 | 3-Aminoisobutanoate | 2 | GMD |
| 471 | C02612 | Citramalate | 1 | STD in this study |
| 474 | C00153 | Nicotinamide | 2 | MSDIAL-DB |
| 478 | C00149 | Malate | 2 | MSDIAL-DB |
| 498 | C00049 | Aspartate | 2 | MSDIAL-DB |
| 499 | C00073 | Methionine | 1 | STD in this study |
| 501 | C01879 | Pyroglutamate | 1 | STD in this study |
| 525 | C01020 | 6-Hydroxynicotinate | 2 | MSDIAL-DB |
| 528 | C02266 | Xylonolactone | 2 | MSDIAL-DB |
| 529 | C02630 | 2-hydroxyglutarate | 2 | MSDIAL-DB |
| 530 | C00026 | 2-Oxoglutarate | 1 | STD in this study |
| 538 | C02504 | 2-Isopropylmalate | 2 | GMD |
| 540 | C00103 | Glucose-1P | 1 | STD in this study |
| 561 | C00025 | Glutamate | 1 | STD in this study |
| 567 | C00156 | 4-Hydroxybenzoate | 2 | MSDIAL-DB |
| 571 | C00079 | Phenylalanine | 2 | MSDIAL-DB |
| 599 | C21057 | Ribose | 2 | MSDIAL-DB |
| 627 | C00379 | Xylitol | 2 | MSDIAL-DB |
| 637 | C00417 | *Cis*-Aconitate | 2 | MSDIAL-DB |
| 641 | C00134 | Putrescine | 2 | MSDIAL-DB |
| 643 | C00628 | Gentisate | 2 | MSDIAL-DB |
| 659 | C00064 | Glutamine | 2 | MSDIAL-DB |
| 676 | C00363 | Thymidine 5'-diphosphate (dTDP) | 1 | STD in this study |
| 685 | C00197 | 3-phosphoglycerate | 1 | STD in this study |
| 692 | C00158 | Citrate | 2 | MSDIAL-DB |
| 697 | C07326 | 1,5-Anhydro-D-glucitol | 2 | MSDIAL-DB |
| 710 | C00147 | Adenine | 2 | MSDIAL-DB |
| 726 | C00031 | Glucose | 2 | MSDIAL-DB |
| 730 | C00047 | Lysine | 2 | MSDIAL-DB |
| 735 | C00082 | Tyrosine | 1 | STD in this study |
| 740 | C02262 | Galactosamine | 2 | MSDIAL-DB |
| 751 | C00864 | Pantothenate | 2 | MSDIAL-DB |
| 774 | C01419 | Cysteinylglycine | 2 | MSDIAL-DB |
| 779 | C00355 | Levodopa | 2 | MSDIAL-DB |
| 799 | C00117 | Ribose 5-phosphate | 1 | STD in this study |
| 817 | C00051 | Glutathione | 2 | MSDIAL-DB |
| 838 | C00315 | Spermidine | 2 | MSDIAL-DB |
| 847 | C02291 | Cystathionine | 2 | MSDIAL-DB |
| 851 | C00092 | Glucose-6P | 2 | MSDIAL-DB |
| 857 | C01096 | Sorbitol-6P | 2 | MSDIAL-DB |
| 895 | C01083 | Trehalose | 2 | MSDIAL-DB |
| 912 | C00008 | Adenosine 5'-diphosphate (ADP) | 2 | MSDIAL-DB |

^§^Confidence level based on MSI (1); ^#^Combined database from MSDIAL project aligned to Kovats retention index (2, 3); *standard compounds.

Table S3: ANOVA results assessed the statistical variances of metabolite features between strains BL:Veb, BL:VbT2, BL:CpVe, and BL:CpT2. *P*-values are FDR corrected.

| Alignment ID | *P*-value |  | Alignment ID | *P*-value |
| --- | --- | --- | --- | --- |
| 18 | 1.83E-01* |  | 599 | 1.35E-07 |
| 63 | 7.68E-01 |  | 600 | 1.34E-03 |
| 82 | 4.98E-01 |  | 601 | 1.34E-03 |
| 94 | 9.16E-02 |  | 602 | 1.34E-03 |
| 127 | 5.46E-01 |  | 603 | 3.61E-07 |
| 130 | 4.97E-04 |  | 613 | 4.79E-01 |
| 196 | 8.58E-04 |  | 615 | 7.76E-01 |
| 197 | 8.58E-04 |  | 623 | 2.23E-06 |
| 213 | 8.36E-12 |  | 626 | 4.34E-09 |
| 218 | 4.60E-02 |  | 627 | 4.45E-13 |
| 222 | 9.60E-01 |  | 629 | 2.97E-03 |
| 237 | 4.82E-01 |  | 630 | 2.97E-03 |
| 238 | 4.82E-01 |  | 632 | 2.52E-01 |
| 240 | 2.35E-01 |  | 633 | 2.33E-08 |
| 241 | 2.35E-01 |  | 637 | 3.53E-04 |
| 247 | 2.79E-05 |  | 639 | 2.69E-01 |
| 252 | 2.96E-01 |  | 640 | 2.69E-01 |
| 253 | 6.85E-04 |  | 641 | 2.69E-01 |
| 266 | 7.92E-03 |  | 643 | 9.50E-06 |
| 269 | 1.36E-05 |  | 649 | 3.16E-13 |
| 270 | 1.36E-05 |  | 653 | 8.90E-02 |
| 272 | 1.27E-02 |  | 654 | 5.27E-02 |
| 276 | 3.81E-01 |  | 655 | 5.27E-02 |
| 279 | 3.49E-01 |  | 656 | 1.90E-05 |
| 282 | 3.31E-06 |  | 657 | 5.27E-02 |
| 283 | 2.13E-03 |  | 659 | 3.06E-01 |
| 284 | 9.89E-02 |  | 664 | 4.28E-10 |
| 286 | 4.68E-01 |  | 665 | 1.26E-02 |
| 287 | 4.68E-01 |  | 670 | 1.71E-10 |
| 288 | 4.68E-01 |  | 672 | 8.89E-03 |
| 289 | 4.68E-01 |  | 674 | 3.13E-07 |
| 307 | 1.11E-02 |  | 675 | 1.70E-02 |
| 310 | 9.96E-01 |  | 676 | 8.21E-03 |
| 312 | 4.68E-01 |  | 677 | 4.28E-10 |
| 320 | 1.70E-02 |  | 678 | 1.86E-06 |
| 322 | 1.99E-04 |  | 679 | 2.74E-06 |
| 323 | 6.49E-06 |  | 684 | 5.80E-01 |
| 327 | 5.34E-02 |  | 685 | 5.80E-01 |
| 328 | 5.34E-02 |  | 686 | 5.59E-01 |
| 332 | 6.69E-01 |  | 688 | 3.05E-01 |
| 339 | 4.85E-04 |  | 689 | 7.26E-07 |
| 340 | 6.10E-02 |  | 690 | 4.09E-01 |
| 342 | 7.96E-02 |  | 691 | 9.14E-02 |
| 347 | 9.64E-02 |  | 692 | 9.16E-02 |
| 354 | 8.89E-02 |  | 693 | 4.68E-01 |
| 356 | 8.82E-02 |  | 697 | 2.15E-04 |
| 359 | 6.04E-02 |  | 700 | 1.16E-04 |
| 360 | 9.89E-02 |  | 701 | 1.55E-01 |
| 368 | 6.70E-02 |  | 702 | 2.96E-01 |
| 369 | 3.97E-02 |  | 703 | 2.94E-01 |
| 376 | 1.27E-01 |  | 706 | 5.97E-01 |
| 377 | 1.70E-01 |  | 710 | 4.24E-01 |
| 381 | 4.39E-07 |  | 715 | 1.13E-03 |
| 384 | 4.80E-01 |  | 720 | 3.16E-13 |
| 385 | 1.53E-03 |  | 724 | 6.43E-01 |
| 394 | 4.26E-02 |  | 725 | 6.40E-01 |
| 398 | 4.67E-01 |  | 726 | 1.44E-05 |
| 401 | 1.83E-02 |  | 728 | 6.95E-01 |
| 411 | 3.64E-01 |  | 729 | 3.59E-01 |
| 412 | 4.63E-01 |  | 730 | 3.46E-01 |
| 419 | 8.00E-01 |  | 731 | 3.46E-01 |
| 421 | 1.48E-01 |  | 735 | 9.09E-01 |
| 422 | 1.21E-02 |  | 737 | 9.09E-01 |
| 423 | 7.75E-01 |  | 740 | 3.29E-04 |
| 425 | 1.26E-12 |  | 742 | 2.32E-01 |
| 427 | 2.37E-02 |  | 750 | 1.35E-05 |
| 428 | 1.24E-10 |  | 751 | 4.79E-01 |
| 432 | 2.99E-01 |  | 752 | 5.73E-02 |
| 433 | 2.24E-03 |  | 753 | 2.49E-03 |
| 434 | 3.31E-02 |  | 755 | 3.27E-01 |
| 435 | 2.47E-02 |  | 761 | 2.38E-02 |
| 441 | 2.09E-02 |  | 762 | 4.45E-13 |
| 442 | 3.29E-04 |  | 769 | 4.28E-01 |
| 447 | 1.27E-01 |  | 770 | 1.86E-06 |
| 456 | 2.56E-02 |  | 771 | 1.39E-03 |
| 460 | 1.53E-03 |  | 774 | 3.95E-02 |
| 462 | 1.37E-10 |  | 775 | 3.97E-02 |
| 465 | 4.69E-02 |  | 776 | 6.15E-02 |
| 467 | 1.98E-02 |  | 777 | 1.91E-02 |
| 468 | 4.46E-01 |  | 778 | 2.64E-01 |
| 469 | 5.94E-01 |  | 779 | 2.39E-02 |
| 470 | 7.61E-01 |  | 780 | 9.02E-01 |
| 471 | 1.98E-02 |  | 781 | 5.46E-01 |
| 473 | 1.49E-11 |  | 782 | 3.64E-01 |
| 474 | 6.60E-02 |  | 783 | 3.88E-05 |
| 475 | 6.60E-02 |  | 791 | 5.24E-04 |
| 478 | 8.82E-02 |  | 793 | 3.23E-07 |
| 483 | 3.22E-01 |  | 795 | 5.61E-07 |
| 492 | 9.87E-04 |  | 799 | 1.86E-01 |
| 498 | 2.28E-02 |  | 802 | 2.96E-01 |
| 499 | 2.36E-01 |  | 804 | 6.31E-03 |
| 501 | 1.61E-01 |  | 806 | 7.78E-19 |
| 508 | 1.79E-03 |  | 807 | 9.27E-14 |
| 509 | 1.78E-03 |  | 808 | 2.77E-01 |
| 513 | 2.65E-01 |  | 809 | 4.10E-07 |
| 517 | 1.93E-02 |  | 810 | 2.66E-01 |
| 524 | 8.73E-02 |  | 811 | 2.24E-07 |
| 525 | 1.98E-02 |  | 813 | 1.08E-08 |
| 528 | 7.13E-04 |  | 814 | 4.25E-04 |
| 529 | 1.86E-01 |  | 817 | 1.44E-01 |
| 530 | 2.00E-02 |  | 819 | 7.00E-09 |
| 531 | 2.04E-02 |  | 822 | 5.07E-01 |
| 533 | 2.59E-09 |  | 832 | 3.91E-01 |
| 534 | 3.48E-09 |  | 836 | 1.27E-01 |
| 537 | 5.59E-01 |  | 837 | 6.64E-02 |
| 538 | 9.06E-03 |  | 838 | 4.33E-01 |
| 539 | 9.25E-03 |  | 839 | 3.69E-01 |
| 540 | 4.40E-05 |  | 842 | 9.05E-02 |
| 549 | 3.73E-08 |  | 843 | 7.06E-03 |
| 552 | 2.72E-05 |  | 847 | 4.56E-06 |
| 553 | 1.90E-05 |  | 851 | 4.06E-03 |
| 554 | 1.90E-05 |  | 852 | 6.88E-02 |
| 555 | 2.55E-01 |  | 854 | 6.78E-03 |
| 556 | 1.90E-05 |  | 856 | 1.27E-01 |
| 560 | 2.67E-02 |  | 857 | 1.27E-01 |
| 561 | 2.67E-02 |  | 862 | 2.04E-02 |
| 562 | 1.90E-05 |  | 864 | 1.53E-02 |
| 564 | 2.67E-02 |  | 869 | 8.58E-04 |
| 566 | 4.19E-04 |  | 877 | 2.24E-07 |
| 567 | 4.97E-04 |  | 880 | 1.16E-01 |
| 568 | 3.06E-01 |  | 894 | 1.34E-05 |
| 571 | 5.72E-01 |  | 895 | 1.34E-05 |
| 572 | 1.39E-01 |  | 897 | 1.18E-01 |
| 580 | 1.62E-05 |  | 898 | 1.70E-02 |
| 581 | 5.72E-05 |  | 911 | 2.04E-01 |
| 583 | 6.55E-08 |  | 912 | 2.50E-01 |
| 587 | 6.43E-02 |  | 913 | 1.13E-01 |
| 597 | 5.22E-07 |  | 914 | 2.77E-01 |
| 598 | 3.35E-07 |  |  |  |

*Numbers represented in scientific mode; for example, 3.35E-07 represents a scientific number as 3.35x10^-07^.

Table S4. T-test results by comparing pairs of variants. The t-test results for the 30 annotated metabolites that were highlighted by ANOVA are listed below. Metabolites that were significantly increased (*P*-value < 0.05 and Log_2_(FC) > 1) are highlighted in red, while those significantly decreased (*P*-value < 0.05 and Log_2_(FC) < -1) are in blue. *P*-values are FDR corrected.

| **KEGG ID** | **Metabolites** | **Alignment ID** | **BL:CpVe vs. BL:Veb** | | **BL:VbT2 vs. BL:Veb** | | **BL:CpT2 vs. BL:VeT2** | |
| --- | --- | --- | --- | --- | --- | --- | --- | --- |
|  |  |  | Log_2_(FC) | *P*-value | Log_2_(FC) | *P*-value | Log_2_(FC) | *P*-value |
| C00022 | Pyruvate | 197 | -0.3108 | 0.4146 | 2.1170 | 0.0018 | -2.1882 | 0.0034 |
| C00186 | Lactate | 213 | -1.6719 | 0.1026 | 4.6887 | 7.92E-05 | -6.2495 | 9.26E-09 |
| C00160 | Glycolate | 218 | 0.4332 | 0.1080 | -0.0686 | 0.8170 | 0.6778 | 0.0773 |
| C00189 | Ethanolamine | 339 | 1.1444 | 0.0037 | 1.2502 | 0.0004 | -0.1694 | 0.5376 |
| C00258 | Glycerate | 394 | 0.9255 | 0.0446 | 0.6520 | 0.0230 | -0.0119 | 0.9719 |
| C00122 | Fumarate | 401 | 1.6025 | 0.0717 | -0.4043 | 0.3593 | 1.6419 | 0.0554 |
| C00178 | Thymine | 433 | 0.6056 | 0.0095 | 0.1522 | 0.3593 | 0.4830 | 0.0591 |
| C05145 | 3-Aminoisobutanoate | 465 | 1.0769 | 0.0897 | 0.1263 | 0.6144 | 1.0956 | 0.0888 |
| C00815 | Citramalate | 471 | 0.7932 | 0.7828 | 2.5193 | 0.0007 | -0.8067 | 0.1665 |
| C00049 | Aspartate | 498 | 1.1999 | 0.0191 | 0.3585 | 0.6144 | 0.9261 | 0.1707 |
| C01020 | 6-Hydroxynicotinate | 525 | 1.4107 | 0.0321 | 0.7073 | 0.0183 | 0.7264 | 0.2749 |
| C02266 | Xylonolactone | 528 | 2.3094 | 0.0144 | 0.7807 | 0.1151 | 1.7470 | 0.0174 |
| C00026 | 2-Oxoglutarate | 530 | 0.2609 | 0.5594 | 2.1354 | 0.0048 | -1.2511 | 0.1330 |
| C02504 | 2-Isopropylmalate | 538 | 1.2175 | 0.1045 | 1.7282 | 0.0006 | -0.0748 | 0.7314 |
| C00103 | Glucose-1P | 540 | 0.8375 | 0.0014 | 0.4222 | 0.0147 | 0.3715 | 0.0609 |
| C00025 | Glutamate | 561 | 3.0523 | 0.1027 | 3.0231 | 0.0024 | 0.4683 | 0.8714 |
| C00156 | 4-Hydroxybenzoate | 567 | 0.0815 | 0.7379 | 0.8700 | 0.0006 | -0.8011 | 0.0082 |
| C00121 | Ribose | 599 | 2.3036 | 0.0231 | -5.2767 | 0.0394 | 7.7403 | 2.06E-08 |
| C00379 | Xylitol | 627 | 3.1603 | 2.39E-08 | 0.2083 | 0.1844 | 2.8851 | 1.44E-06 |
| C00417 | Cis-Aconitate | 637 | -0.7586 | 0.1990 | 1.3544 | 0.0058 | -2.0518 | 0.0012 |
| C00628 | Gentisate | 643 | 1.9009 | 0.0024 | -0.1109 | 0.8336 | 1.6250 | 0.0006 |
| C00363 | dTDP | 676 | 1.8838 | 0.0295 | 0.3503 | 0.3412 | 1.5806 | 0.0557 |
| C07326 | 1,5-Anhydro-D-glucitol | 697 | 1.3824 | 0.0033 | 0.7190 | 0.1119 | 0.7404 | 0.0590 |
| C00031 | Glucose | 726 | 0.8098 | 0.0829 | 4.0172 | 0.0013 | -2.9843 | 0.0088 |
| C02262 | Galactosamine | 740 | 1.7043 | 0.0036 | 0.7087 | 0.1536 | 0.9329 | 0.0609 |
| C01419 | Cysteinylglycine | 774 | 5.0378 | 0.0463 | 2.3861 | 0.2849 | 2.8741 | 0.1665 |
| C00355 | Levodopa | 779 | -0.2255 | 0.4280 | 0.7552 | 0.0229 | -0.7219 | 0.0788 |
| C00542 | Cystathionine | 847 | 6.2584 | 0.0015 | -0.1391 | 0.6869 | 6.6117 | 0.0005 |
| C00092 | Glucose-6P | 851 | 1.8817 | 0.0144 | 2.1872 | 0.0020 | -0.2311 | 0.6433 |
| C01083 | Trehalose | 895 | 2.1042 | 0.0208 | 6.4082 | 0.0006 | -4.0660 | 0.0148 |

Table S5. Detailed results of the pathway analysis with 30 affected metabolites. *P*-values are FDR corrected.

| **Pathways** | **Hit/Total** | ***P*-value** | **Impact** |
| --- | --- | --- | --- |
| Alanine, aspartate and glutamate metabolism | 5/22 | 7.24E-03 | 0.531 |
| Arginine biosynthesis | 4/16 | 1.40E-02 | 0.102 |
| TCA cycle | 4/20 | 1.71E-02 | 0.133 |
| Glyoxylate and dicarboxylate metabolism | 5/37 | 1.71E-02 | 0.159 |
| Starch and sucrose metabolism | 4/22 | 1.71E-02 | 0.536 |
| Pyruvate metabolism | 4/27 | 3.25E-02 | 0.366 |
| Butanoate metabolism | 3/18 | 7.44E-02 | 0.169 |
| Valine, leucine and isoleucine biosynthesis | 3/22 | 1.01E-01 | 0.164 |
| Monobactam biosynthesis | 2/8 | 1.01E-01 | 0.000 |
| C5-Branched dibasic acid metabolism | 2/8 | 1.01E-01 | 0.000 |
| Pentose phosphate pathway | 3/26 | 1.40E-01 | 0.000 |
| Glycolysis/Gluconeogenesis | 3/29 | 1.73E-01 | 0.080 |
| Glycine, serine and threonine metabolism | 3/33 | 2.26E-01 | 0.021 |
| Pentose and glucuronate interconversions | 3/38 | 3.03E-01 | 0.000 |
| Galactose metabolism | 3/39 | 3.03E-01 | 0.051 |
| Cysteine and methionine metabolism | 3/41 | 3.04E-01 | 0.003 |
| Carbapenem biosynthesis | 1/3 | 3.04E-01 | 0.000 |
| Acarbose and validamycin biosynthesis | 1/3 | 3.04E-01 | 0.000 |
| Glutathione metabolism | 2/22 | 3.79E-01 | 0.069 |
| Pantothenate and CoA biosynthesis | 2/24 | 4.10E-01 | 0.000 |
| Polyketide sugar unit biosynthesis | 1/5 | 4.10E-01 | 0.000 |
| D-Amino acid metabolism | 2/25 | 4.10E-01 | 0.174 |
| Lipoic acid metabolism | 2/28 | 4.75E-01 | 0.000 |
| Dioxin degradation | 1/7 | 4.97E-01 | 0.000 |
| Xylene degradation | 1/7 | 4.97E-01 | 0.000 |
| Aminobenzoate degradation | 1/8 | 5.41E-01 | 0.000 |
| Phenylalanine metabolism | 2/35 | 5.83E-01 | 0.000 |
| Nitrogen metabolism | 1/11 | 6.70E-01 | 0.000 |
| Lysine biosynthesis | 1/13 | 7.18E-01 | 0.000 |
| beta-Alanine metabolism | 1/13 | 7.18E-01 | 0.000 |
| Amino sugar and nucleotide sugar metabolism | 2/44 | 7.18E-01 | 0.080 |
| Benzoate degradation | 1/15 | 7.26E-01 | 0.000 |
| Glycerolipid metabolism | 1/15 | 7.26E-01 | 0.000 |
| Nicotinate and nicotinamide metabolism | 1/15 | 7.26E-01 | 0.000 |
| Cyanoamino acid metabolism | 1/17 | 7.62E-01 | 0.000 |
| Pyrimidine metabolism | 2/51 | 7.62E-01 | 0.030 |
| Terpenoid backbone biosynthesis | 1/18 | 7.77E-01 | 0.000 |
| Ascorbate and aldarate metabolism | 1/20 | 7.96E-01 | 0.013 |
| O-antigen nucleotide sugar biosynthesis | 1/21 | 7.96E-01 | 0.159 |
| Valine, leucine and isoleucine degradation | 1/22 | 7.96E-01 | 0.000 |
| Glycerophospholipid metabolism | 1/22 | 7.96E-01 | 0.030 |
| Lysine degradation | 1/22 | 7.96E-01 | 0.118 |
| Thiamine metabolism | 1/23 | 7.96E-01 | 0.000 |
| Ubiquinone and other terpenoid-quinone biosynthesis | 1/23 | 7.96E-01 | 0.057 |
| Methane metabolism | 1/26 | 8.54E-01 | 0.000 |
| Arginine and proline metabolism | 1/29 | 9.04E-01 | 0.048 |
| Porphyrin metabolism | 1/35 | 1.00E+00 | 0.000 |

**Supplementary References:**

1. Sumner LW, Amberg A, Barrett D, Beale MH, Beger R, Daykin CA, et al. Proposed minimum reporting standards for chemical analysis Chemical Analysis Working Group (CAWG) Metabolomics Standards Initiative (MSI). Metabolomics: Official journal of the Metabolomic Society. 2007;3(3):211-21.

2. Tsugawa H, Cajka T, Kind T, Ma Y, Higgins B, Ikeda K, et al. MS-DIAL: data-independent MS/MS deconvolution for comprehensive metabolome analysis. Nat Methods. 2015;12(6):523-6.

3. Kovats E, Giddings JC, Keller R. Gas chromalographic characterization of organic substances in the retention index system. Advances in Chromatography. 1965;1:229-47.
